# Defining blood-induced microglia functions in neurodegeneration through multiomic profiling

**DOI:** 10.1038/s41590-023-01522-0

**Published:** 2023-06-08

**Authors:** Andrew S. Mendiola, Zhaoqi Yan, Karuna Dixit, Jeffrey R. Johnson, Mehdi Bouhaddou, Anke Meyer-Franke, Min-Gyoung Shin, Yu Yong, Ayushi Agrawal, Eilidh MacDonald, Gayathri Muthukumar, Clairice Pearce, Nikhita Arun, Belinda Cabriga, Rosa Meza-Acevedo, Maria del Pilar S. Alzamora, Scott S. Zamvil, Alexander R. Pico, Jae Kyu Ryu, Nevan J. Krogan, Katerina Akassoglou

**Affiliations:** 1 Gladstone Institutes, San Francisco, CA USA; 2 Center for Neurovascular Brain Immunology at Gladstone and UCSF, San Francisco, CA USA; 3 Department of Microbiology, Immunology and Molecular Genetics, University of California, Los Angeles, CA USA; 4 Quantitative Biosciences Institute, University of California, San Francisco, CA USA; 5 Department of Neurology, Weill Institute for Neurosciences, University of California, San Francisco, CA USA; 6 Department of Cellular and Molecular Pharmacology, University of California, San Francisco, CA USA

**Keywords:** Neuroimmunology, Innate immunity

## Abstract

Blood protein extravasation through a disrupted blood–brain barrier and innate immune activation are hallmarks of neurological diseases and emerging therapeutic targets. However, how blood proteins polarize innate immune cells remains largely unknown. Here, we established an unbiased blood-innate immunity multiomic and genetic loss-of-function pipeline to define the transcriptome and global phosphoproteome of blood-induced innate immune polarization and its role in microglia neurotoxicity. Blood induced widespread microglial transcriptional changes, including changes involving oxidative stress and neurodegenerative genes. Comparative functional multiomics showed that blood proteins induce distinct receptor-mediated transcriptional programs in microglia and macrophages, such as redox, type I interferon and lymphocyte recruitment. Deletion of the blood coagulation factor fibrinogen largely reversed blood-induced microglia neurodegenerative signatures. Genetic elimination of the fibrinogen-binding motif to CD11b in Alzheimer’s disease mice reduced microglial lipid metabolism and neurodegenerative signatures that were shared with autoimmune-driven neuroinflammation in multiple sclerosis mice. Our data provide an interactive resource for investigation of the immunology of blood proteins that could support therapeutic targeting of microglia activation by immune and vascular signals.

## Abstract (web summary)

Akassoglou and colleagues provide a single-cell RNA sequencing and phosphoprotein analysis of the responses of central nervous system microglia and macrophages to blood proteins including activated complement and fibrin. Their findings point to potential therapeutic targeting of microglia activation by immune and vascular signals.

## Main

Vascular and immune signals are potent activators of the innate immune response in a wide range of autoimmune, inflammatory and infectious diseases in the brain and the periphery^1–4^. Innate immune cells integrate environmental signals to rapidly activate target genes and perform specialized cellular functions^5^. Pathogenic activation of microglia contributes to oxidative stress, inflammation and neurodegeneration in both Alzheimer’s disease (AD) and multiple sclerosis (MS)^6^. Blood–brain barrier (BBB) disruption is an early pathological feature linked to microglial activation, neurodegeneration and progression in MS, AD and other neurological diseases^3,7^. Upon BBB disruption, toxic blood proteins extravasate into the brain, altering the environmental milieu^7–10^. Blood coagulation and complement pathways are key activators of innate immunity that are coregulated in aging, cancer, and neurological, psychiatric and infectious diseases^11–17^. The blood coagulation protein fibrinogen is converted to insoluble fibrin at sites of vascular damage and induces microglia activation and neurodegeneration via the CD11b–CD18 integrin receptor (also known as complement receptor 3 (CR3), α_M_β_2_ or Mac-1)^3,18–21^. CR3 also binds additional structurally unrelated ligands with diverse immunomodulatory functions, including complement protein iC3b, which regulates synaptic, pathogen and debris clearance via phagocytosis^1,22^. However, how microglia integrate extracellular signals at sites of cerebrovascular damage and the specificity of blood proteins controlling innate immune cell polarization in disease remain poorly understood.

We report a blood-induced microglia gene network and show that blood proteins elicit distinct receptor-mediated transcriptional changes and signaling programs in innate immune cells. We provide a transcriptomic and phosphoproteomic atlas of fibrin-, iC3b- and lipopolysaccharide (LPS)-selective activation of innate immunity and reveal ligand-selective pathways with differential functions in MS and AD mice. We identify fibrin–CD11b signaling as causal for neurotoxic microglial programming in disease. Moreover, our study provides a resource for the investigation of the immunology of blood proteins in inflammatory, autoimmune and neurodegenerative diseases.

## Results

### Blood-induced microglial transcriptomic profiling

**Fig. 1:**
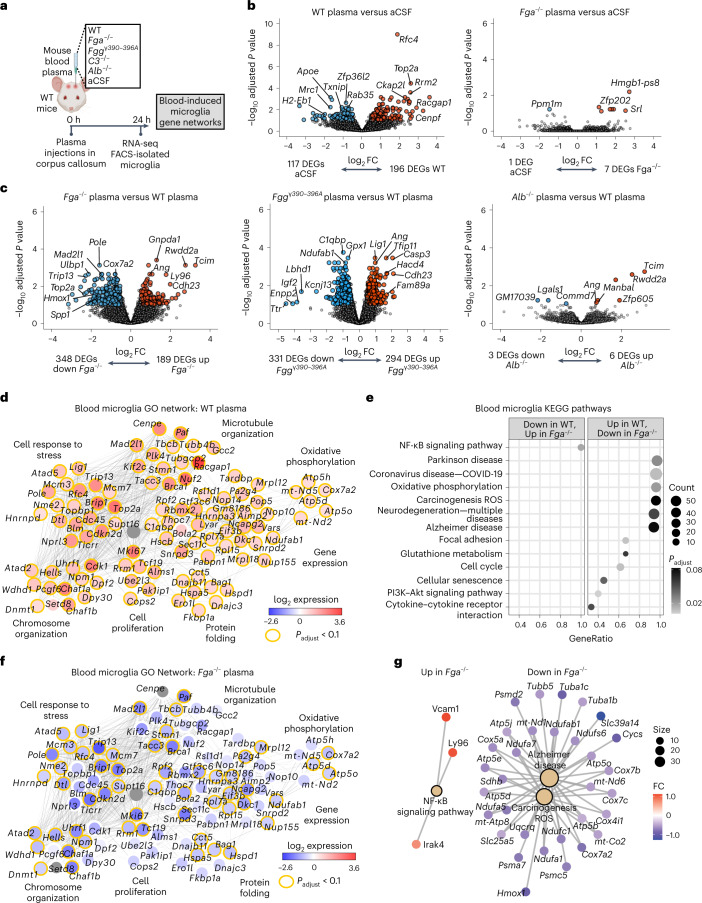
Transcriptional profiling of ligand-selective activation of blood-induced microglial responses in vivo. **a**, Schematic of experimental design for transcriptional profiling of blood-induced microglial responses. **b**,**c**, Volcano plots of DEGs from RNA-seq analysis of sorted microglia from plasma-injected brains. Comparisons between DEGs in microglia of brains injected with WT plasma versus aCSF or *Fga*^*−/*^^*−*^ plasma versus aCSF (**b**) and *Fga*^*−/*^^*−*^ plasma versus WT plasma, *Fgg*^*γ390*–^^*396A*^ versus WT plasma or *Alb*^*−/*^^*−*^ versus WT plasma (**c**) are shown. The log_2_ FC and −log_10_ adjusted *P* value cutoffs were log_2_ FC > 0.5, adjusted *P* < 0.1 with Wald test followed by Benjamini–Hochberg (BH) test correction. Top DEGs are shown. Data are from *n* = 6 *Fga*^*−/*^^*−*^, *n* = 6 WT, *n* = 6 aCSF and *n* = 8 *Alb*^*−/*^^*−*^ mice. **d**, Coexpression GO networks upregulated in microglia by WT plasma. Adjusted *P* value <0.1 by hypergeometric test and BH test correction. **e**, GSEA plots of top upregulated and downregulated pathways in microglia from *Fga*^*−/*^^*−*^ plasma-injected versus WT plasma-injected brains. Adjusted *P* value <0.1 by permutation test with BH test correction. **f**, Overlay of blood microglia GO network with microglial gene expression values from *Fga*^*−/*^^*−*^ plasma-injected mice. Red shading, genes upregulated in microglia by WT plasma; blue shading, genes downregulated in microglia by *Fga*^*−/*^^*−*^ plasma; orange border, *P < 0.1 (Wald test followed by BH test correction). **g**, Coexpression KEGG pathway networks of top upregulated and downregulated pathways in microglia from *Fga*^*−/*^^*−*^ plasma-injected versus WT plasma-injected brains. Adjusted *P* < 0.1 by hypergeometric distribution and BH test correction. *P*_adjust_, adjusted *P* value. Created with BioRender.com.

**Extended Data Fig. 1:**
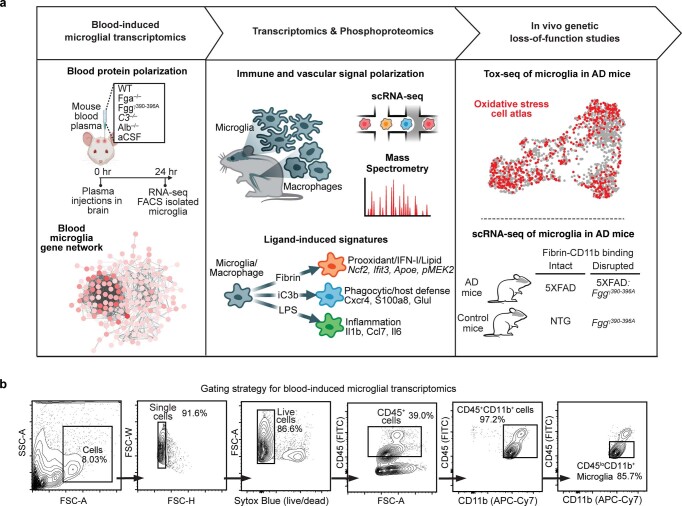
Blood-innate immunity multiomics pipeline. **a**, Blood-innate immunity multiomics pipeline for identification of blood-induced immune pathways and in vivo validation studies. **b**, Gating strategy for blood-induced microglia transcriptomic profiling.

**Extended Data Fig. 2:**
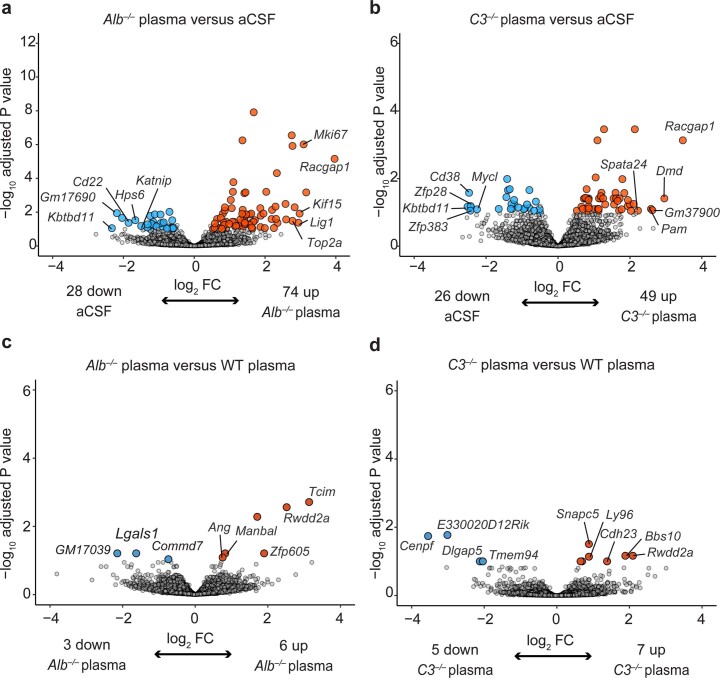
RNA-seq analysis of microglia from plasma injected brains. **a-d**, Volcano plots of DEGs from RNA-seq analysis of sorted microglia from plasma or aCSF injected brains. Comparisons between DEGs in microglia from *Alb*^*–/–*^ plasma vs aCSF (**a**), *C3*^*–/–*^ plasma vs aCSF (**b**), *Alb*^*–/–*^ plasma vs WT plasma (**c**) or *C3*^*–/–*^ plasma vs WT plasma (**d**) injected brains are shown. Dots depict average log_2_FC and -log_10_ adjusted *P* values (abs(log_2_ > 0.5, adjusted *P* value < 0.1 with Wald test followed by Benjamini-Hochberg multiple test correction). Data are from *n* = 6 *Fga*^*–/–*^, *n* = 6 WT, *n* = 6 aCSF, *n* = 8 *Alb*^*–/–*^ mice.

**Extended Data Fig. 3:**
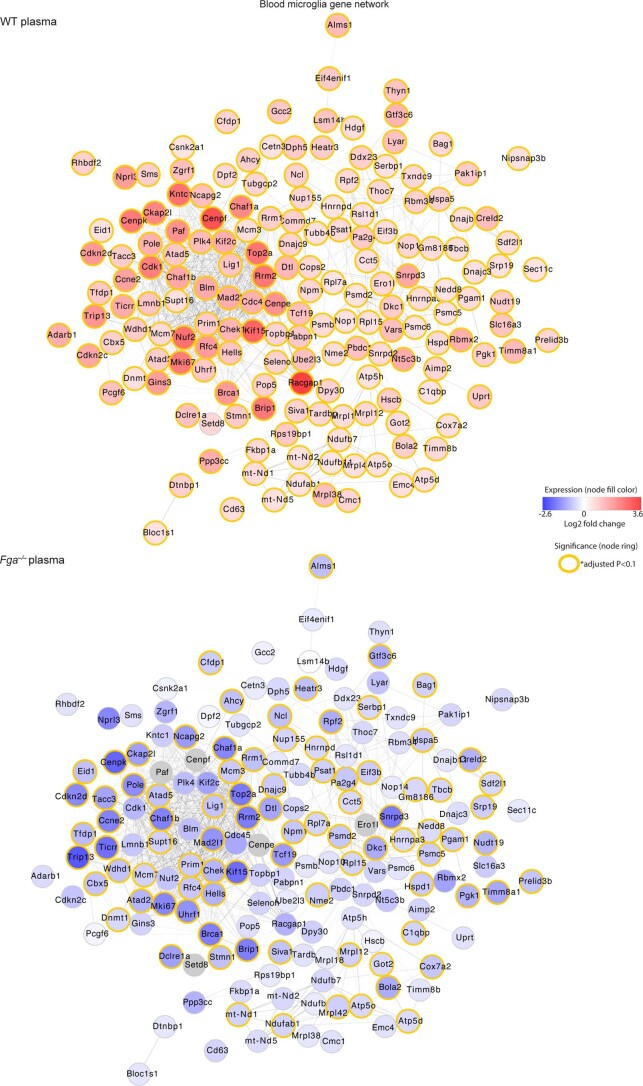
Blood-induced microglial gene co-expression network. Top, Co-expression gene network of 196 DEGs upregulated in microglia by WT plasma compared to aCSF control. Bottom, overlay of blood gene network with microglial gene expression values from Fga^–/–^ plasma-treated mice. Red shading, genes upregulated in microglia; blue shading, genes downregulated in microglia; the orange boarder indicates significance of *P* < 0.1 (Wald test following by Benjamini-Hochberg multiple test correction). Data are from *n* = 3 biological replicates per group, with two mice pooled per replicate.

To discover the molecular programs controlling microglial and macrophage polarization by blood proteins, we developed an unbiased blood-innate immunity multiomic and genetic loss-of-function pipeline consisting of deep sequencing of blood-induced transcriptomes, functional single-cell and oxidative stress transcriptomics, global phosphoproteomics and integration with innate immune signatures from AD and MS models (Extended Data Fig. 1). To determine the blood-induced transcriptome in microglia, we stereotactically delivered wild-type (WT) plasma to the brain, followed by RNA sequencing (RNA-seq) analysis of sorted microglial cells (Fig. 1a and Supplementary Table 1). By unbiased analyses of differentially expressed genes (DEGs), gene ontology (GO) networks and Kyoto Encyclopedia of Genes and Genomes (KEGG) pathways, we showed that WT plasma induced widespread microglial transcriptional changes, including changes involving genes related to oxidative stress (for example, *Hmox1*, *Romo1, Gpx1)*, disease-associated microglia (DAM) (for example, *Cst7*, *Spp1*) and the cell cycle (for example, *Top2a*, *Cdkn2d*), as well as reactive oxygen species (ROS), oxidative phosphorylation, neurodegeneration, AD, glutathione metabolism and COVID-19 pathways (Fig. 1b–e and Supplementary Table 2). These changes were largely absent following stimulation with plasma derived from fibrinogen-deficient *Fga*^−^^/−^ mice but were relatively preserved following stimulation with complement 3-deficient (*C3*^*−/*^^*−*^) or albumin-deficient (*Alb*^*−/*^^*−*^) plasma (Fig. 1b,c, Extended Data Fig. 2 and Supplementary Tables 2 and 3). Indeed, the number of genes in the blood-induced microglia gene network was reduced by 97%, with significant downregulation of 52% of the genes, when plasma was derived from *Fga*^*−/*^^*−*^ mice (Fig. 1f, Extended Data Fig. 3 and Supplementary Table 3), suggesting that fibrinogen is a key protein in the blood that induces microglia activation. Through unbiased KEGG pathway analysis of DEGs between *Fga*^*−/*^^*−*^ and WT plasma-stimulated microglia, we identified the 12 top pathways induced by fibrinogen, including ROS (for example, *Hmox1*, *Cox7a2*, *Slc25a5*), COVID-19 (for example, *Ccl12*, *Rps8*, *Rpl35*) and AD (for example, *Atp5e*, *Psmd2*, *Tubb5*) (Fig. 1e,g). Similarly, microglia gene expression was reduced in response to plasma from *Fgg*^*γ390*–^^*396A*^ mice, in which fibrinogen had been mutated to lack the CD11b–CD18 binding motif but retained normal clotting function^19,23^ compared with WT plasma (Fig. 1c and Supplementary Table 2). Whereas the effect of *C3*^*−/*^^*−*^ or *Alb*^*−/*^^*−*^ plasma on microglia was largely similar to that of WT plasma (five and three DEGs, respectively), *Fga*^*−/*^^*−*^ and *Fgg*^*γ390*–*396A*^ plasma induced major gene expression changes in microglia (348 and 331 DEGs, respectively) (Fig. 1c, Extended Data Fig. 2 and Supplementary Table 2). These results are consistent with reduced demyelination in the corpus callosum induced by *Fga*^*−/*^^*−*^ or *Fgg*^*γ390*–^^*396A*^ plasma compared with WT plasma or fibrinogen administration^21,24^. Collectively, these results suggest that there is specificity among blood proteins in the induction of microglia transcriptional changes, indicating that fibrinogen signaling is a critical regulator in the blood for the induction of oxidative stress and disease-induced signatures in microglia following BBB leakage.

### Single-cell RNA-seq reveals distinct fibrin and iC3b gene signatures

**Fig. 2:**
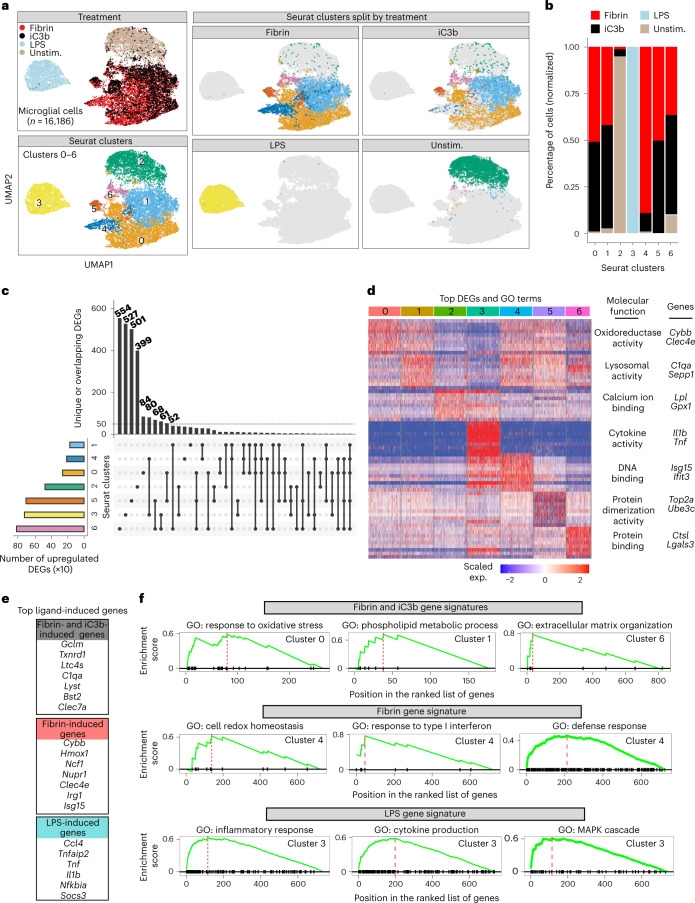
scRNA-seq analysis of ligand-selective activation of microglia. **a**, UMAP plots of single microglial cells treated with fibrin, iC3b and LPS identified by unsupervised clustering analysis (*n* = 16,196 cells from two independent experiments). **b**, Fractions of cells in each Seurat cluster colored based on treatment label. **c**, UpSet plot showing a matrix layout of DEGs specific to a cluster (single filled circle with no vertical lines) and DEGs shared between clusters (filled circles connected with vertical lines). Vertical bar plot of the unique or overlapping DEGs in clusters (top). Horizontal bar plot of the number of upregulated DEGs for a cluster (left). **d**, Heat map of top ten DEGs per single-cell cluster. Example GO molecular function terms and genes for a given cluster are shown. Five-hundred cells (maximum) were randomly selected from each cluster, as shown in **a**, for visualization. Gene expression is depicted as log-normalized scaled expression. **e**, List of selected top upregulated ligand-induced genes. **f**, GSEA plots of top GO terms for ligand-induced gene signatures. Adjusted *P* < 0.05 by Kolmogorov–Smirnov test with BH test correction. Exp., expression; unstim., unstimulated.

**Extended Data Fig. 4:**
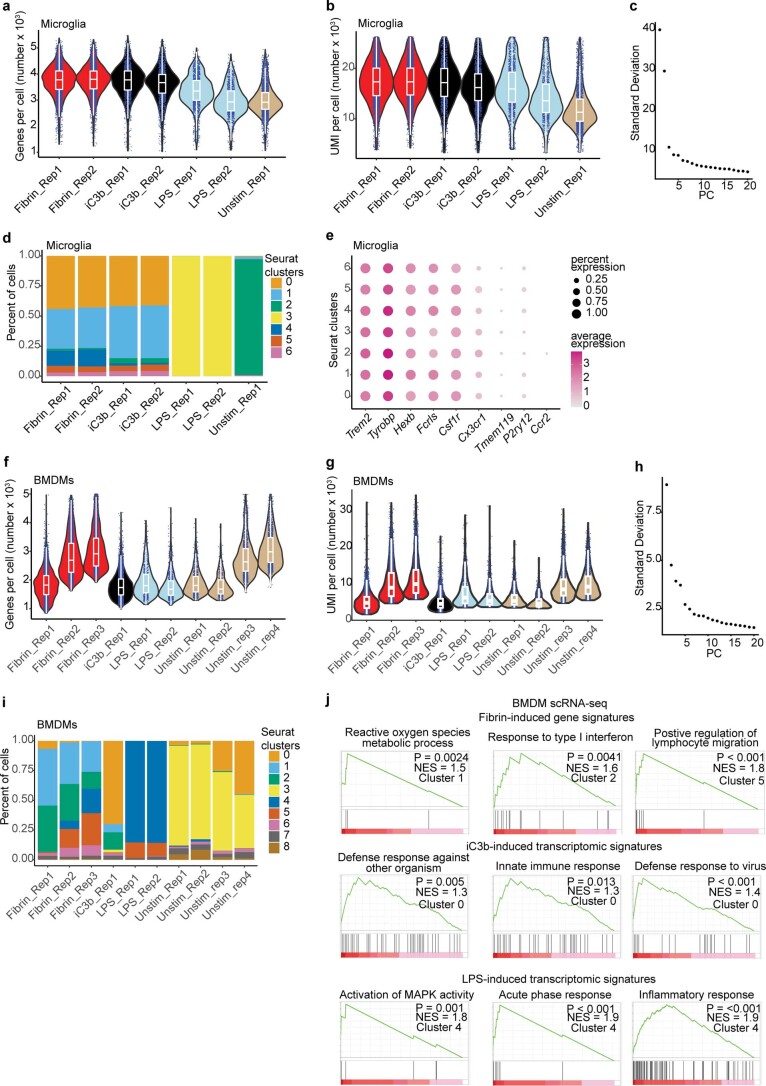
Quality control for primary microglia scRNA-seq and BMDM scRNA-seq datasets. **a, b**, Violin plots of number genes (**a**) and unique molecular identifiers (UMI, **b**) per cell post-normalization and shown for each biologically independent sample from scRNA-seq of primary microglia stimulated with fibrin, iC3b, LPS or left unstimulated. Data are from two biologically independent samples of fibrin, two biologically independent samples of iC3b, two biologically independent samples of LPS, and one biologically independent sample of unstimulated primary microglia. **c**, Elbow plot of top PC used to select for clustering analysis. **d**, Distribution of cells in each biologically independent sample across each seurat clusters. **e**, Dot plot of selected microglial gene markers across each cluster from scRNA-seq dataset of primary microglia as shown in Fig. 3a. Average gene expression and cell population expression is depicted as log expression and percent, respectively. **f, g**, Violin plots of number genes (**f**) and UMI (**g**) per cell post-normalization and shown for each biologically independent sample from scRNA-seq of primary BMDMs stimulated with fibrin, iC3b, LPS or left unstimulated. Data are from three biologically independent samples of fibrin, one biologically independent sample of iC3b, two biologically independent samples of LPS and four biologically independent samples of unstimulated BMDMs. **h**, Elbow plot of top PC used to select for clustering analysis. **i**, Distribution of cells in each biologically independent sample across each seurat clusters. **j**, Gene-set enrichment plots of top GO terms for a given cluster (adjusted *P* value < 0.05 with BH correction). Violin plots depict minimum, maximum, and median expression, with points showing single-cell expression levels (**a**, **b, f, g**). Box plots show the 1st to 3rd quartiles (25–75% box bounds) with median values indicated and upper and lower whiskers extending to 1.5*inter-quartile range (**a**, **b, f, g**).

To determine how innate immune cells polarize in response to immune and vascular signals, we first generated single-cell RNA-seq (scRNA-seq) profiles from fibrin-, complement iC3b- or LPS-stimulated primary microglia (Fig. 2a–e and Extended Data Fig. 4). Gene set enrichment analysis (GSEA) of top DEGs identified fibrin-induced genes controlling oxidative stress and redox regulation (for example, *Cybb*, *Ncf1*, *Clec4e*) and the type 1 interferon (IFN)-stimulated gene (ISG) family (for example, *Isg15*, *Ifit3* and *Ifit2*), which were selectively enriched in cluster 4 ‘Mg-fibrin-cluster’ (Fig. 2f). iC3b-treated cells overlapped with fibrin-treated cells (clusters 0, 1, 5 and 6; here termed ‘fibrin-iC3b-clusters’) and were characterized by 1,184 coregulated genes, with 111 genes (~9%) overlapping with LPS-induced gene response (Fig. 2c,d and Supplementary Table 4). Both fibrin and iC3b induced common gene signatures related to pathways of oxidative stress (for example, *Hmox1*, *Prdx6* and *Txnrd1*), phospholipid metabolism (for example, *C1qa*, *Sepp1* and *Clec7a*) and organization of extracellular matrix (ECM) (for example, *Ctsl*, *Lgals3* and *Apoe*) (Fig. 2f and Supplementary Table 4). As expected, LPS activated gene expression programs of the inflammatory response including mitogen-activated protein kinase (MAPK) activity in microglia (Fig. 2f and Supplementary Table 4). Overall, these results highlight ligand-selective gene circuits in microglia.

**Fig. 3:**
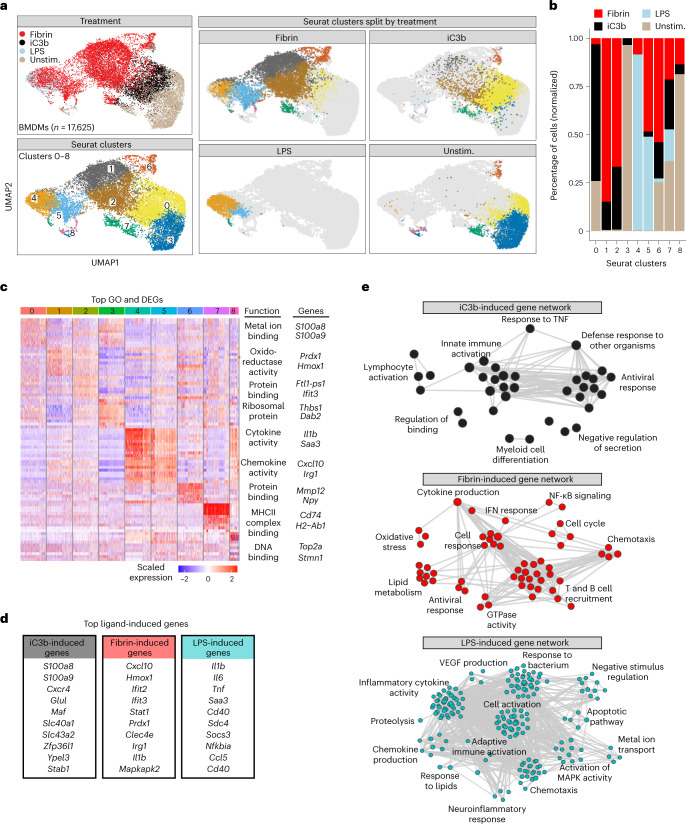
Single-cell RNA-seq analysis of ligand-selective activation of macrophages. **a**, UMAP plots of single BMDMs treated with fibrin, iC3b or LPS identified by unsupervised clustering analysis (*n* = 17,625 cells from six mice (fibrin), two mice (iC3b), four mice (LPS) and eight mice (unstimulated)). BMDMs are shown colored by treatment, Seurat cluster or both. **b**, Fraction of cells in each Seurat cluster colored based on treatment label. **c**, Heat map of top ten DEGs per single-cell cluster. Example GO molecular function terms and genes for a given cluster are shown. Five-hundred cells (maximum) were randomly selected from each cluster, as shown in **a**, for visualization. Gene expression is depicted as log-normalized scaled expression. **d**, List of selected top upregulated ligand-induced genes. **e**, Coexpression GO term networks for iC3b (black-filled nodes), fibrin (red-filled nodes) and LPS (cyan-filled nodes) from scRNA-seq of BMDMs. Upregulated GO terms are shown as colored nodes, and gene coexpression overlap is shown as gray edges. *P* < 0.10 (fibrin and iC3b) and *P* < 0.01 (LPS) by GSEA Kolmogorov–Smirnov test without multiple test correction.

**Extended Data Fig. 5:**
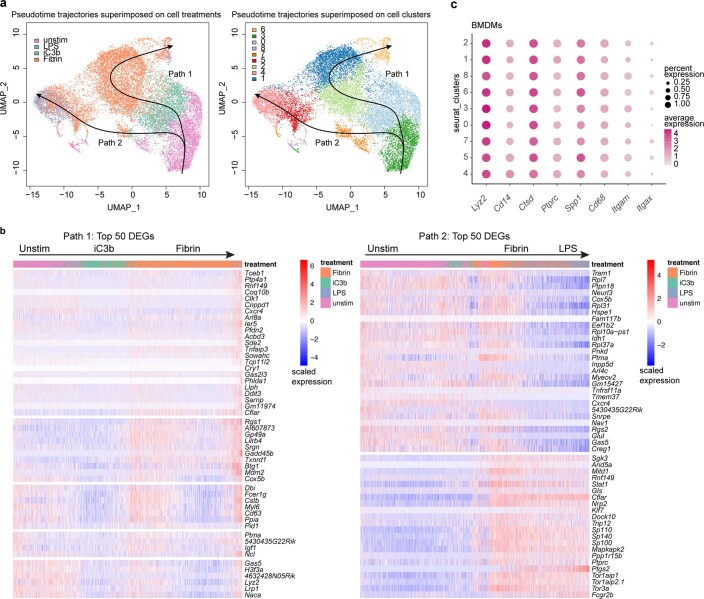
Pseudotime trajectory analysis of scRNA-seq BMDM profiles. **a**, UMAP plots of BMDM scRNA-seq profiles colored by treatment (left) or cluster (right) as shown in Fig. 3a, overlaid with pseudotime trajectory analysis with Slingshot. Black arrows indicate inferred trajectories with unstimulated (cluster 3) defined as starting point. **b**, Heat map of single cell gene expression patterns across pseudotime trajectories of top 50 DEGs. **c**, Dot plot of selected macrophage gene markers across each cluster from scRNA-seq datasets of primary BMDMs as shown in Fig. 3a. Average gene expression and cell population expression is depicted as log expression and percent, respectively.

Next, we performed a similar analysis on mouse bone-marrow-derived macrophages (BMDMs), unstimulated or treated with either fibrin, complement iC3b or LPS. Like microglia, BMDMs largely clustered into distinct ligand-induced transcriptional states, with clusters enriched for fibrin (clusters 1, 2, 5 and 6; termed ‘BMDM-fibrin-clusters’), iC3b (cluster 0; termed ‘BMDM-complement-cluster’) and LPS (clusters 4 and 5; termed ‘BMDM-LPS-clusters’) (Fig. 3a–d, Extended Data Fig. 4f–i and Supplementary Table 5). Similar to stimulated microglia, GSEA identified fibrin-induced genes *Cxcl10*, *Hmox1*, *Ifit3*, *Prdx1*, *Clec4e*, *Nos2*, *Il1b* and *Stat1* linked with oxidative stress, IFN-I response, lipid metabolism, and T and B cell recruitment (Fig. 3d,e and Extended Data Fig. 4j, Supplementary Table 5). By contrast, GO analysis of the BMDM-complement-cluster showed enrichment of pathways for host defense response, myeloid cell differentiation, innate immune and lymphocyte activation and of related genes (for example, *S100a8*, *S100a9*, *Cxcr4*, *Glul* and *Maf*) (Fig. 3d,e and Extended Data Fig. 4j). Antiviral gene signatures were identified as shared GO terms in both the fibrin and iC3b clusters (Fig. 3e and Extended Data Fig. 4l). As expected for LPS-primed macrophages^5^, LPS clusters were enriched in gene pathways for MAPK activity, inflammatory cytokine and chemokine production, and chemotaxis (for example, *Il1b*, *Il6* and *Tnf*) (Fig. 3d, Extended Data Fig. 4j and Supplementary Table 5). Pseudotime analysis showed a two-path transcriptional bifurcation from unstimulated to iC3b to the fibrin-induced state (path 1) or unstimulated to fibrin to the LPS-induced state (path 2) (Extended Data Fig. 5a,b and Supplementary Table 6), suggesting that the CR3 and TLR4 ligands induce distinct activation pathways. All cell clusters were enriched for monocyte-derived macrophage markers (for example, *Lyz2*, *Cd14* and *Ctsd*) and had low expression of monocyte-derived dendritic cell markers (for example, *Itgax*) (Extended Data Fig. 5c), suggesting that undifferentiated or contaminating cells were not a major driver of clustering. Together, these results show that fibrin polarization primarily promotes prooxidant, lipid metabolism and IFN-I responses, whereas complement iC3b and LPS induce host defense and classical inflammatory states, respectively.

### Phosphoproteomics reveal distinct fibrin and iC3b signaling

**Extended Data Fig. 6:**
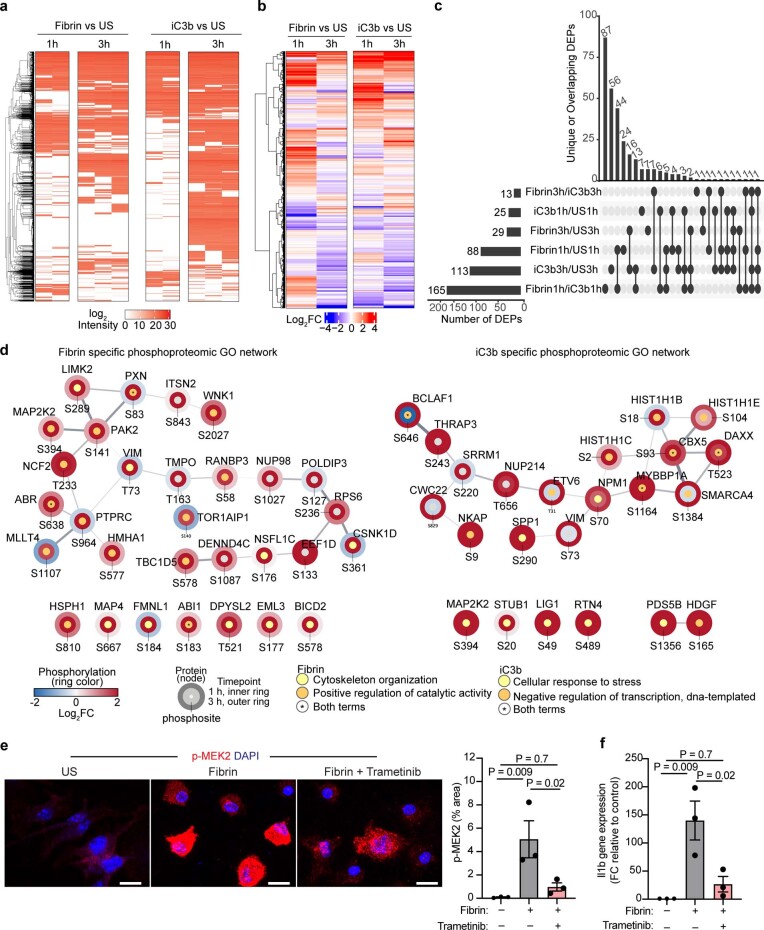
Network analysis and functional validation of phoshoproteomic dataset. **a**, Log_2_ intensity of phosphorylated peptides (rows) for each biological replicate (columns) across stimulation comparisons at each time point. Cells colored white indicate not detected. Unstimulated, US. Data from *n* = 2 (Fibrin 1 h), *n* = 2 (iC3b 1 h), *n* = 3 (US 1 h), *n* = 3 (Fibrin 3 h), *n* = 3 (iC3b 3 h), *n* = 3 (US 3 h) independent experiments. **b**, Log2FC of phosphorylated peptides between fibrin vs US and iC3b vs US at each timepoint. **c**, UpSet plot showing a matrix layout of DEPs specific to a treatment (single filled circle with no vertical lines) or shared between treatment (filled circles connected with vertical lines) comparisons. Bar plots of the unique or overlapping DEPs in treatment comparison (top) and the number DEPs for each treatment comparison (left). Phosphorylation sites considered DEPs (FDR < 0.05 and abs(log_2_ FC) > 1.5) are shown. Student’s t-test with BH correction. **d**, Phosphoproteomic GO network for fibrin or iC3b. Phosphorylation changes (log_2_ FC) depicted as blue-red scheme and timepoint (h) as rings. GO terms indicated by node fill color and protein interaction strength as edge thickness and opacity. **e**, Confocal microscopy of p-MEK2 staining in BMDMs left unstimulated or stimulated for 90 min with fibrin alone or in the presence of trametinib. Nuclei labeled with DAPI. Scale bar, 50 μm. Quantification of p-MEK2 for *n* = 3 independent experiments in duplicates. *P* < 0.05 as determined by two-tailed unpaired t-test. **f**, Quantitative PCR of *Il1b* expression in BMDMs unstimulated or stimulated for 6 h with fibrin alone or in the presence of trametinib. Data are from *n* = 3 independent experiments performed in duplicates. *P* < 0.05 as determined by one-way ANOVA with Tukey’s multiple comparisons test. Source data

Although macrophage signal transduction pathways have been extensively studied for Toll-like receptor ligands^25^, the downstream signaling cascades for CR3 ligand activation have not been characterized. We performed unbiased quantitative phosphoproteomics^26^ using mass spectrometry to globally characterize the dynamics of protein phosphorylation in response to complement iC3b or fibrin stimulation in RAW 264.7 macrophages. Hierarchical clustering analysis of detected phosphorylation sites revealed distinct signaling profiles for fibrin and iC3b (Extended Data Fig. 6a,b and Supplementary Tables 7 and 8). Fibrin initially induced a greater increase in global phosphorylation relative to iC3b, as evidenced by a significant increase in the detection and abundance of differentially expressed phosphosites (DEPs) 1 h after treatment (Extended Data Fig. 6a–c and Supplementary Table 9). iC3b stimulation induced a greater number of detected phosphosites at 3 h after stimulation (Extended Data Fig. 6a–c and Supplementary Table 9), suggesting differential phosphorylation kinetics.

**Fig. 4:**
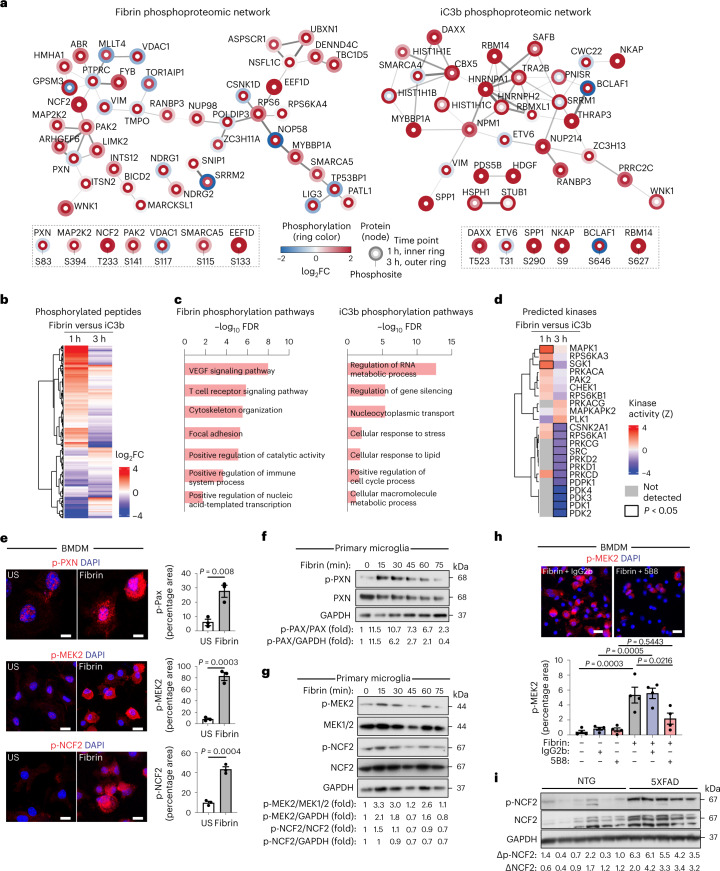
Phosphoproteomics of fibrin and iC3b signaling in innate immune cells. **a**, Phosphoproteomic interaction networks for fibrin and iC3b. Selected phosphopeptides shown with phosphorylation as blue–red scheme and time points as rings. Adjusted *P* < 0.05. **b**, DEPs between fibrin and iC3b at each time point. **c**, Functional enrichment terms from fibrin or iC3b phosphorylation pathways. **d**, Kinase activities from the phosphoproteomic dataset. Differentially regulated kinases between fibrin and iC3b are indicated with black bounding. Kinases below the cutoff are in gray. **e**, Confocal microscopy and quantification of p-MEK2, p-PXN and p-NCF2 staining in BMDMs stimulated with fibrin or unstimulated (US). **f**,**g**, Immunoblot of p-PXN, PXN and GAPDH (**f**) and p-MEK2, MEK1/2, p-NCF2 and GAPDH (**g**) in primary rat microglia, US or stimulated with fibrin; FC compared with control is indicated. **h**, Microscopy and quantification of p-MEK2 staining in BMDMs left US or stimulated for 90 min with fibrin alone or in the presence of 5B8 or IgG2b. **i**, Immunoblot of p-NCF2, NCF2 and GAPDH from 12-month-old 5XFAD or NTG mouse cortex. Signal ratios (delta) for p-NCF2–GAPDH and NCF2–GAPDH are shown. Data are from *n* = 2 (fibrin 1 h), *n* = 2 (iC3b 1 h), *n* = 3 (US 1 h), *n* = 3 (fibrin 3 h), *n* = 3 (iC3b 3 h) and *n* = 3 (US 3 h) independent experiments (**a**–**d**); *n* = 3 independent experiments in duplicate (**e**), representative of two independent experiments (**f**,**g**); *n* = 4 independent experiments in duplicates (**h**); or *n* = 6 (NTG) and *n* = 5 (5XFAD) mice (**i**). Statistics: two-sided Student’s *t* test with BH test correction (**a** and **b**), FDR < 0.05 by hypergeometric test with BH correction (**c**), *P* < 0.05 by two-sided *z* test (**d**), two-tailed unpaired *t* test (**e**) or one-way ANOVA with Tukey’s multiple comparisons test (**h**). Data are mean ± s.e.m., and nuclei are labeled with DAPI (**e** and **h**). Scale bars, 10 μm (**e**); 50 μm (**h**). US, unstimulated. Source data

Next, we generated the fibrin and iC3b phosphoproteomic interaction networks. Fibrin and iC3b induced unique and dynamic phosphorylation events, with few DEPs shared among ligands (Fig. 4a,b and Supplementary Tables 8 and 9). Fibrin induced robust phosphorylation of proteins including integrin-associated focal adhesion adaptor protein paxillin (PXN), nicotinamide adenine dinucleotide phosphate (NADPH) oxidase subunit neutrophil cytosolic factor 2 (NCF2), and mitochondria oxidative phosphorylation and metabolic function voltage-dependent anion-selective channel protein 1 (VDAC1) (Fig. 4a and Supplementary Table 8). Fibrin induced phosphorylation of Cdc42/Rac-activated serine/threonine protein kinase 2 (PAK2), which links Rho GTPases to cytoskeleton reorganization and nuclear signaling, and redox regulators SWI/SNF-related matrix-associated actin-dependent regulator of chromatin subfamily A member 5 (SMARCA5) and elongation factor 1-delta (EEF1D) (Fig. 4a and Supplementary Table 8). Fibrin also induced phosphorylation of IFN regulatory factor 2 binding protein 2 (IRF2BP2), which is a transcriptional cofactor inducing VEGF expression and angiogenesis. iC3b induced phosphorylation of death domain-associated protein (DAXX), secreted phosphoprotein 1 (SPP1, also known as osteopontin), ETS variant transcription factor 6 (ETV6) and STIP1 homology and U-Box containing protein 1 (STUB1) (Fig. 4a and Supplementary Table 8). Fibrin and iC3b induced phosphorylation of MAP2K2 (also known as MEK2), NF-κB-activating protein (NKAP) and Ran-binding protein 3 (RANBP3) (Fig. 4a and Supplementary Table 8). String functional enrichment identified differential pathways induced by fibrin including ‘VEGF signaling’, ‘T cell receptor signaling’, ‘cytoskeleton organization’ and ‘focal adhesion’ and by iC3b including ‘regulation of RNA metabolic process’, ‘regulation of gene silencing’ and ‘cellular response to stress’ (Fig. 4c and Supplementary Table 10). Phosphoproteomic GO pathway interaction networks showed that fibrin induced dynamic regulation of ‘cytoskeleton organization’ and ‘positive regulation of catalytic activity’ networks, whereas iC3b led to sustained activation of ‘cellular response to stress’ and ‘negative regulation of transcription, DNA-templated’ interaction networks (Extended Data Fig. 6d and Supplementary Table 10).

The regulation of phosphorylation signaling cascades is largely mediated by protein kinases. To predict kinase–substrate relationships, we bioinformatically calculated kinase activities from our phosphoproteomics data^26^. We identified significant activation of MAPK1 (also known as extracellular signal-regulated kinase 2, ERK2) and serum-glucocorticoid kinase 1 (SGK1) at 1 h after fibrin but not iC3B treatment (Fig. 4d and Supplementary Table 11). At 3 h after stimulation, we observed significant activation of casein kinase II (CSNK2A1), S6 kinase (RPS5KA1), protein kinase C and D kinases, and pyruvate dehydrogenase kinases (PDK1–4) in iC3b-treated samples relative to fibrin-treated samples (Fig. 4d and Supplementary Table 11). Together, these results show that fibrin and iC3b induce differential phosphorylation events and kinase activities, suggesting that blood proteins induce distinct signal transduction pathways in innate immune cells.

**Extended Data Fig. 7:**
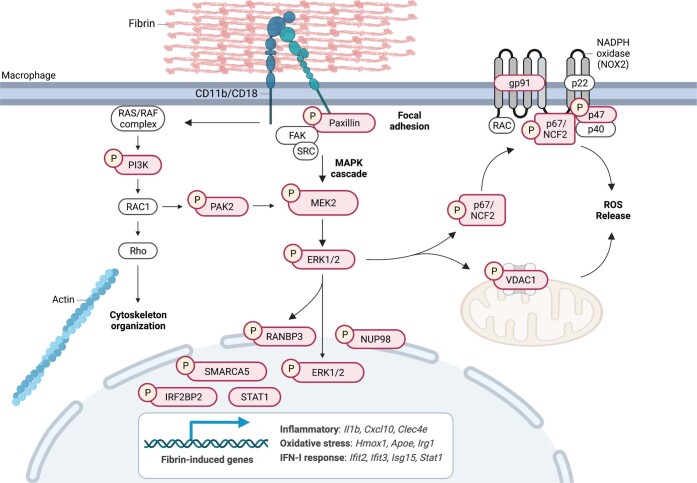
A model of fibrin-induced signal transduction in macrophages. Fibrin binding to CD11b-CD18 leads to the conversion of the integrin to the high-affinity extended-open (active) conformation that induces signal transduction in macrophages. This outside-in signaling is propagated by the formation of focal adhesions through recruitment and phosphorylation of scaffold proteins and signaling kinases such as paxillin and focal adhesion kinase (FAK) resulting in phosphorylation of PI3K and cytoskeleton organization. In parallel, the MAPK cascade MEK2 and ERK1/2 components are phosphorylated leading to 1) transactivation of NADPH oxidase complex (NOX2) and mitochondria responses to induce ROS release and oxidative stress, 2) phosphorylation of SMARCA5, NUP98, and the ERK1/2 nuclear transporter RANBP3 to regulate nuclear import, 3) phosphorylation of IRF2B2 regulating IFN signaling and 4) transcriptional activation of fibrin-induced genes involved in inflammatory, oxidative stress, and IFN-I responses. Phosphorylation (P); fibrin-induced proteins identified in this study, red filled shapes; fibrin-induced genes identified in this study are shown in box. Created with Biorender.com.

Next, we validated the top proteins phosphorylated by fibrin in BMDMs, primary microglia and brain tissue from AD mice. Paxillin binds to the cytoplasmic domain of β2 subunits of integrins including CD11b–CD18, and its phosphorylation initiates focal adhesion complex formation upon integrin engagement with ECM^27^. Fibrin induced dynamic phosphorylation of paxillin at residue 83 (p-PXN) in BMDMs and primary microglia (Fig. 4e,f), consistent with fibrin activation of focal adhesions in platelets^28^. MEK2 phosphorylates ERK1/2, resulting in increased cellular proliferation and migration, oxidative stress and inflammation. We tested the effects of fibrin on MEK2 phosphorylation and proinflammatory gene activation. Fibrin induced robust phosphorylation of MEK2 at residue 394 (p-MEK2) in BMDMs and microglia (Fig. 4e,g). Specific MEK2 inhibitor trametinib blocked p-MEK2 and reduced expression of fibrin-induced gene *Il1b* in fibrin-treated BMDMs (Extended Data Fig. 6e,f), suggesting that MEK2 activation mediates fibrin-induced proinflammatory gene expression. Similarly, treatment with the therapeutic monoclonal 5B8 antibody, which targets the fibrin-binding site to the CD11b I-domain without affecting fibrin polymerization^29^, blocked p-MEK2 in fibrin-treated BMDMs (Fig. 4h), suggesting that fibrin-induced phosphorylation is receptor mediated. The cytosolic NADPH oxidase subunit NCF2 translocates to the plasma membrane upon phosphorylation by ERK1/2 and phosphatidylinositol-3-kinase, leading to NADPH oxidase activation and ROS production^30^. Fibrin induced phosphorylation of p-NCF2 in BMDMs and primary microglia (Fig. 4e,g), consistent with fibrin activation of NADPH oxidase and ROS generation^20,29,31^. NADPH oxidase activation has been identified in progressive MS^32^ and has been implicated in neurodegeneration and cognitive impairment in AD mice^10,20^. To test whether NCF2 was phosphorylated in disease models in vivo, we assessed NCF2 expression and activation in the brains of 5XFAD mice, a model of AD. p-NCF2 and total NCF2 were higher in 5XFAD than in nontransgenic (NTG) control mice at 12 months of age (Fig. 4i). Together, these results identify fibrin as a CD11b–CD18 ligand coupling integrin signaling with NADPH oxidase activation (Extended Data Fig. 7). They also reveal the phosphoproteome of fibrin and iC3b and demonstrate the specificity of blood proteins in controlling and integrating innate immune signaling pathways in disease.

### Fibrin drives neurotoxic innate immune programs in MS mice

**Fig. 5:**
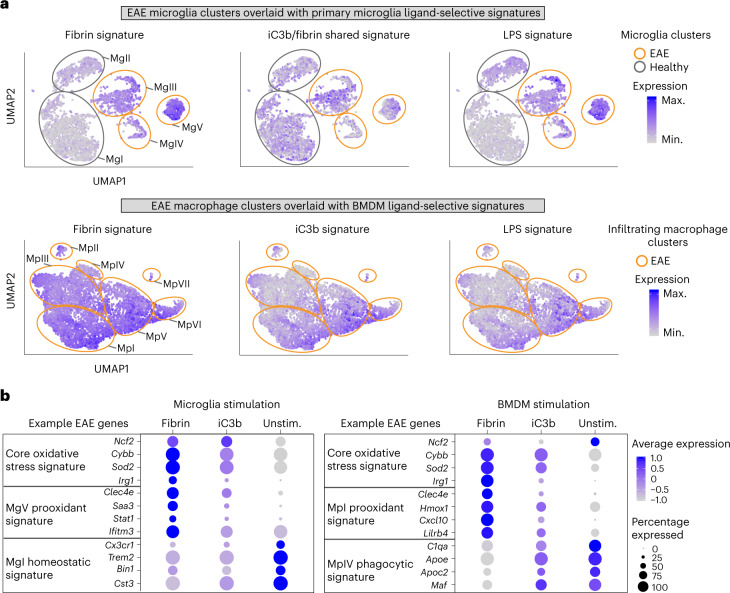
Unbiased overlay of ligand-selective gene signatures with innate immune cell subsets in EAE. **a**, UMAP of EAE microglia or macrophage Tox-seq clusters^31^ overlaid with primary microglia (top) or BMDM (bottom) ligand-activation signatures (this study). Expression is depicted as a log-normalized average modular score for each signature. CNS innate immune cell clusters are numerically labeled and outlined to depict healthy and EAE samples (gray and orange, respectively). **b**, Dot plot of selected gene markers across the scRNA-seq datasets of primary microglia (left panel) and BMDMs (right panel) unstimulated or stimulated with fibrin or iC3b. Average gene expression is depicted as scaled log-normalized expression. Max., maximum; min., minimum.

**Extended Data Fig. 8:**
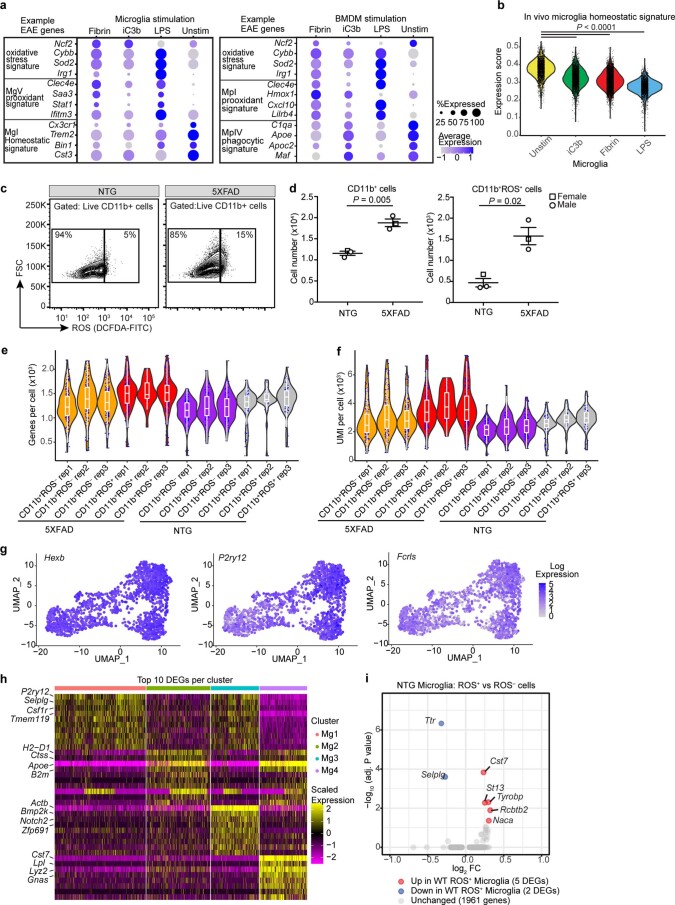
Ligand-induced profile overlays with EAE innate immune cell signatures, and quality control data related to Fig. 6. **a**, Dot plot of selected gene markers across scRNA-seq datasets of primary microglia or BMDMs unstimulated or stimulated with fibrin, iC3b, or LPS. Gene expression is depicted as scaled log-normalized expression. **b**, Violin plot of primary microglia overlaid with microglial homeostatic gene signature from healthy mice as previously identified^31^. Treatments in x-axis are rank ordered from highest to lowest expression. *P* < 0.0001 by one-way ANOVA with Tukey’s multiple comparison test. **c**, Flow cytometry plots of live CD11b^+^ROS^–^ and live CD11b^+^ROS^+^ cells from brains of 12-month-old 5XFAD and NTG mice. Cell population (%) shown inside plot. Data representative of two independent experiments. **d**, Quantification of total live CD11b^+^ cells and CD11b^+^ROS^+^ cells from brains of 12m 5XFAD and NTG mice. Data from *n* = 3 mice per genotype shown as mean ± s.e.m. ROS production assessed via DCFDA (**c**,**d**). *P* < 0.05 as determined by two-tailed, unpaired t-test with Welch’s correction. **e, f**, Violin plots of number genes (**e**) and UMI (**f**) per cell post-normalization, shown for each biologically independent sample for 5XFAD and NTG Tox-seq analysis. Data from *n* = 3 mice per condition. Box plots show the 1st to 3rd quartiles (25–75% box bounds) with median values indicated and upper and lower whiskers extending to 1.5*inter-quartile range. **g**, UMAP plots as shown in Fig. 6a overlaid with microglial gene marker expression. Expression depicted as log-fold change expression. **h**, Heat map of top DEGs per single cell cluster from 5XFAD Tox-seq dataset. Gene expression depicted as scaled z-score. **i**, Volcano plot of DEGs in microglia between CD11b^+^ROS^+^ compared to CD11b^+^ROS^–^ cells in NTG mice. Dots depict average log_2_FC and -log_10_ adjusted *P* values (log_2_ > 0.25, adjusted *P* value < 0.05, MAST test with BH correction). Violin plots depict minimum, maximum, and median expression, with points showing single-cell expression levels (**b**, **e**, **f**). Source data

Oxidative injury is associated with neuronal loss and myelin damage and has been proposed as a key contributor to disease pathogenesis in MS and AD^6,33,34^. As both transcriptomic and phosphoproteomic analyses identified oxidative stress as a key fibrin-induced pathway, we performed an unbiased overlay of the fibrin, iC3b and LPS gene signatures (this study) with oxidative stress central nervous system (CNS) innate immune signatures that have been defined in a model of MS^31^. Single-cell RNA-seq of oxidative stress producing cells (Tox-seq) in an experimental autoimmune encephalomyelitis (EAE) model previously identified distinct cell subsets polarized toward oxidative stress (MgV and MpI clusters), whereas others were enriched in antigen-presenting and phagocytic genes (MgIII and MpIII clusters)^31^. We found that stimulation by fibrin or LPS but not iC3b recapitulated the core oxidative stress signature (for example, *Ncf2*, *Cybb*, *Sod2* and *Irg1*) expressed by ROS^+^ microglia and macrophages in EAE (Fig. 5a,b, Extended Data Fig. 8a and Supplementary Table 12). Fibrin-stimulated cells had the highest expression of prooxidant genes identified in oxidative stress-producing populations of microglia (MgV prooxidant signature *Clec4e*, *Saa3*, *Stat1* and *Ifitm3*) and infiltrating macrophages (MpI prooxidant signature *Clec4e*, *Hmox1*, *Cxcl10* and *Lilrb4*) from EAE (Fig. 5b and Supplementary Table 12). The microglial-shared iC3b–fibrin gene signature (for example, *C1qc*, *C1qa*, *Fcrls*, *Clec7a*, *Apoe*, *Sepp1*) was enriched in the EAE microglia antigen-presenting MgIII cluster^31^ (Fig. 5a,b and Supplementary Table 12). The BMDM iC3b gene signature was enriched in the EAE macrophage clusters MpV and MpVI (Fig. 5a,b), which were identified as phagocytic subsets based on their gene expression programs^31^. All treatments significantly downregulated the homeostatic microglia gene signature (for example, *Cx3cr1*, *Trem2*, *Bin1* and *Cst3*)^31,35^ (Fig. 5b and Extended Data Fig. 8b). The fibrin transcriptomic signature is consistent with reduced oxidative stress, demyelination, axonal damage and protection from paralysis in *Fgg*^*γ390*–^^*396A*^ mice and WT mice treated with fibrin-targeting antibody 5B8 in EAE^19,24,29^. These data suggest that fibrin and complement iC3b are potent signals in neuroinflammatory lesions that recapitulate the polarization of function-specific innate immune responses, with fibrin serving as a potent inducer of oxidative stress gene programs in microglia and peripheral macrophages.

### Fibrin drives neurotoxic microglia programs in AD mice

**Fig. 6:**
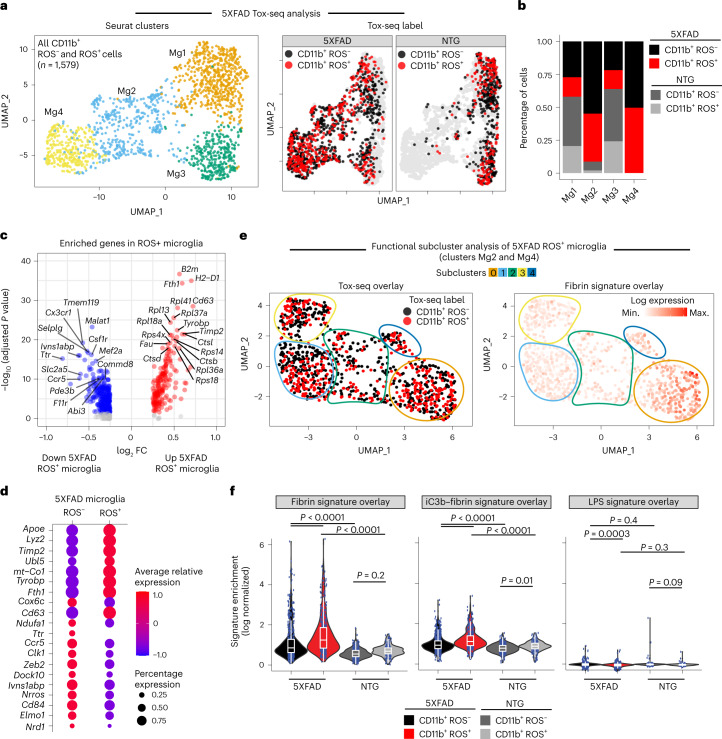
Single-cell oxidative stress transcriptome of microglia in 5XFAD mice. **a**, UMAP plot of single CD11b^+^ ROS^−^ and CD11b^+^ ROS^+^ cells identified by unsupervised clustering analysis (*n* = 1,579 cells from brains of three 5XFAD and three NTG mice) (left). UMAP of Tox-seq-labeled cells from 5XFAD or NTG mice. **b**, Fraction of cells in each Seurat cluster colored based on Tox-seq label. **c**, Volcano plot of enriched DEGs in CD11b^+^ ROS^+^ cells from 5XFAD mice. Dots depict average log_2_ FC and −log_10_ adjusted *P* values (log_2_ FC > 0.25, adjusted *P* < 0.05 with MAST statistical test with BH correction). **d**, Dot plot of selected gene markers from **c**. Average gene expression and cell population expression is depicted as log-normalized scaled expression and percentage, respectively. **e**, UMAP plots of functional subcluster analysis of 5XFAD CD11b^+^ ROS^+^ and CD11b^+^ ROS^−^ microglia overlaid with Tox-seq label (left) or in vitro fibrin signature (right). **f**, Violin plots of fibrin, iC3b–fibrin and LPS gene signature overlays with Tox-seq-labeled microglia. Violin plots depict minimum, maximum and median expression, with points showing single-cell expression levels. Box plots show the first to third quartiles (25–75% box bounds) with median values indicated and upper and lower whiskers extending to 1.5× interquartile range. *n* = 1,579 cells from brains of three 5XFAD and three NTG mice. *P* < 0.05 as determined by two-way ANOVA with Tukey’s multiple comparison test. Source data

We next used Tox-seq to analyze the transcriptomes of CD11b^+^ cells labeled for ROS production (as assessed by 2′,7′-dichlorofluorescein diacetate (DCFDA)) via scRNA-seq from brains of NTG control or 5XFAD mice (Extended Data Fig. 8c–f). Using unbiased clustering analysis superimposed with functional ROS characterization, we identified four transcriptionally distinct CD11b^+^ clusters containing ROS^+^ and ROS^−^ cell populations as visualized by UMAP (Fig. 6a,b and Supplementary Table 13). Most ROS^+^ microglia (50% of cells) from 5XFAD mice were found in microglia cluster 4 (cluster Mg4) (Fig. 6b), which had enrichment of genes involved in neurodegenerative microglial cell activation and iron transport (for example, *Apoe*, *Tyrobp* and *Fth1*) (Fig. 6c,d, Extended Data Fig. 8g,h and Supplementary Table 13). By contrast, genes known to negatively regulate ROS production, superoxide metabolism and maintenance of the microglial homeostatic signature were increased in ROS^−^ microglia from 5XFAD mice (for example, *Nrros*, *Clk1* and *Zeb2*, respectively) (Fig. 6c,d). Differential gene expression analysis showed few changes in ROS^+^ compared with ROS^−^ microglia from NTG mice (Extended Data Fig. 8i). Functional subcluster analysis of both 5XFAD microglia clusters revealed that subcluster 0 had the highest single-cell expression of ROS^+^ microglia (Fig. 6e, Tox-seq cluster overlay). Fibrin-induced genes were enriched in subcluster 0 (Fig. 6e, fibrin signature overlay). ROS^+^ microglia were significantly enriched for fibrin-induced and iC3b–fibrin-induced genes but not LPS-induced genes (Fig. 6f and Supplementary Table 12).

**Fig. 7:**
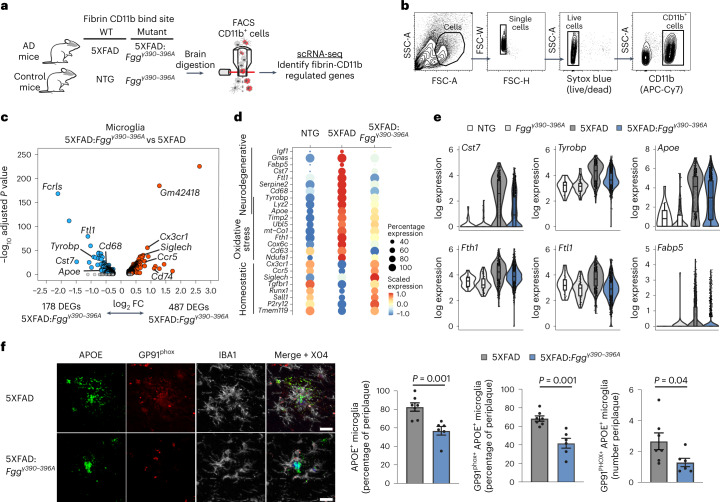
Fibrin induces a neurodegenerative gene signature via CD11b receptor in 5XFAD mice. **a**, Schematic of scRNA-seq analysis of 5XFAD, 5XFAD:*Fgg*^*γ390–396A*^ and control mice. **b**, Gating strategy for brain CD11b^+^ scRNA-seq analysis in mice. **c**, Volcano plot of DEGs in microglia (clusters 1 and 2) between 5XFAD:*Fgg*^*γ390–396A*^ and 5XFAD mice. Dots depict average log_2_ FC and −log_10_ adjusted *P* values (log_2_ FC > 0.25, adjusted *P* < 0.05 with MAST test and BH correction). Data points represent the average of *n* = 3 mice (5XFAD) and *n* = 4 mice (5XFAD:*Fgg*^*γ390–39 6A*^). **d**, Dot plots of microglia neurodegenerative, oxidative stress and homeostatic gene signatures in NTG, 5XFAD and 5XFAD:*Fgg*^*γ390–396A*^ mice. Dot size and color indicate percentage of cells expressing a gene and average expression level, respectively. Data points are averages of *n* = 3 mice (5XFAD) and *n* = 4 mice (5XFAD:*Fgg*^*γ390–396A*^). **e**, Violin plots of log expression levels of microglia genes *Cst7*, *Tyrobp*, *Apoe*, *Fth1*, *Ftl1* and *Fabp5* across genotypes. Violin plots depict minimum, maximum and median expression, with points showing single-cell expression levels. Box plots show the first to third quartiles (25–75% box bounds) with median values indicated and upper and lower whiskers extending to 1.5× interquartile range. Data are from single cells of *n* = 3 mice (NTG), *n* = 4 mice (*Fgg*^*γ390–396A*^), *n* = 3 mice (5XFAD) or *n* = 4 mice (5XFAD:*Fgg*^*γ390–396A*^). **f**, Confocal microscopy images of brain sections from 12-month-old *5XFAD:Fgg*^*γ390–396A*^ and 5XFAD mice immunostained for oxidative stress (GP91^phox^), microglia (IBA1), DAM (APOE) and plaque (Methoxy-X04) markers. Image quantification is shown from *n* = 6 mice (*5XFAD:Fgg*^*γ390–396A*^) and *n* = 7 mice (5XFAD). Data are shown as mean ± s.e.m. *P* < 0.05 by two-tailed Mann–Whitney *U* test. Scale bar, 10 μm. Source data

**Extended Data Fig. 9:**
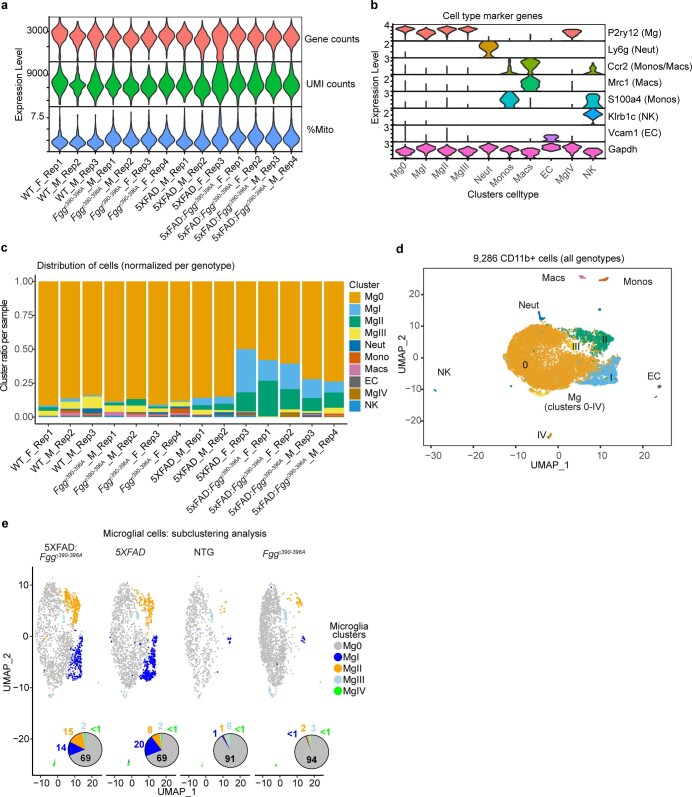
Quality control for scRNA-seq data related to Fig. 7. **a**, Violin plots of gene counts, UMI counts, and %mito for each biologically independent sample. F, female; M, male. **b**, Violin plots of cell type marker genes used to annotate each cluster. **c**, Cluster ratio per biological replicate. **d**, UMAP plot of all single CD11b+ cells identified by clustering analysis (*n* = 9,286 cells from brains of three 5XFAD, three NTG, four 5XFAD*:Fgg*^*γ390-396A*^, and four *Fgg*^*γ390-396A*^ mice). **e**, UMAP plots of microglial cells subclustering analysis split by genotype. Ven diagrams depict percentage of cells per cluster.

To test whether fibrin is necessary for microglial polarization toward neurotoxic phenotypes, we crossed 5XFAD to *Fgg*^*γ390*–^^*396A*^ mice and analyzed microglia by scRNA-seq. The 5XFAD:*Fgg*^*γ390*–^^*396A*^ mice have reduced neurodegeneration and are protected from cognitive impairment^20^. We performed scRNA-seq of CD11b^+^ cells from 5XFAD:*Fgg*^*γ390*–^^*396A*^, 5XFAD, *Fgg*^*γ390*–*396A*^ and NTG littermates (Fig. 7a,b and Extended Data Fig. 9a–d). Differential gene expression analysis of DAM clusters 1 and 2 identified 487 upregulated and 178 downregulated genes in microglia from 5XFAD:*Fgg*^*γ390*^^–^^*39*^^*6A*^ compared with 5XFAD mice (Fig. 7c, Extended Data Fig. 9e and Supplementary Tables 14 and 15). In 5XFAD:*Fgg*^*γ390*^^–^^*39*^^*6A*^ mice, microglia homeostatic genes were among the top upregulated genes (for example, *Cx3cr1*, *Siglech*, *Runx1*), whereas fibrin-induced genes (for example, *Fth1*, *Rnase4*, *Mif*) and known DAM markers (for example, *Cst7*, *Apoe*, *Tyrobp*) were among the top downregulated genes (Fig. 7c and Supplementary Table 15). The neurodegenerative microglia gene signature was significantly reduced in 5XFAD:*Fgg*^*γ390*^^–^^*39*^^*6A*^ mice, whereas microglial homeostatic genes were expressed at control levels (Fig. 7d and Supplementary Table 15). The neurodegenerative signature downregulated in 5XFAD:*Fgg*^*γ390*^^–^^*39*^^*6A*^ mice was related to TREM-2-associated genes^36^ (*Tyrobp*, *CD68*, *Cst7*), iron-binding genes (*Fth1*, *Ftl1*) and lipid-binding genes (*Apoe*, *Fabp5*) (Fig. 7e). To validate these changes in situ, we performed immunohistochemistry in brains from 5XFAD and 5XFAD*:Fgg*^*γ390*^^–^^*39*^^*6A*^ mice using the DAM and oxidative stress markers apolipoprotein E (APOE) and GP91^phox^, which Tox-seq had identified as highly enriched in ROS^+^ microglia. The frequency of double-positive APOE/GP91^phox^ cells surrounding amyloid plaques was reduced in 5XFAD*:Fgg*^*γ390*–^^*396A*^ compared with 5XFAD mice (Fig. 7f), suggesting that *Apoe* gene expression and oxidative-stress-producing DAM around amyloid plaques are fibrin dependent. Overall, these results suggest that fibrin–CD11b signaling drives key microglia pathways including TyroBP, lipid metabolism and oxidative stress responses in neurodegeneration.

### Fibrin microglia signatures shared between AD and MS mice

**Fig. 8:**
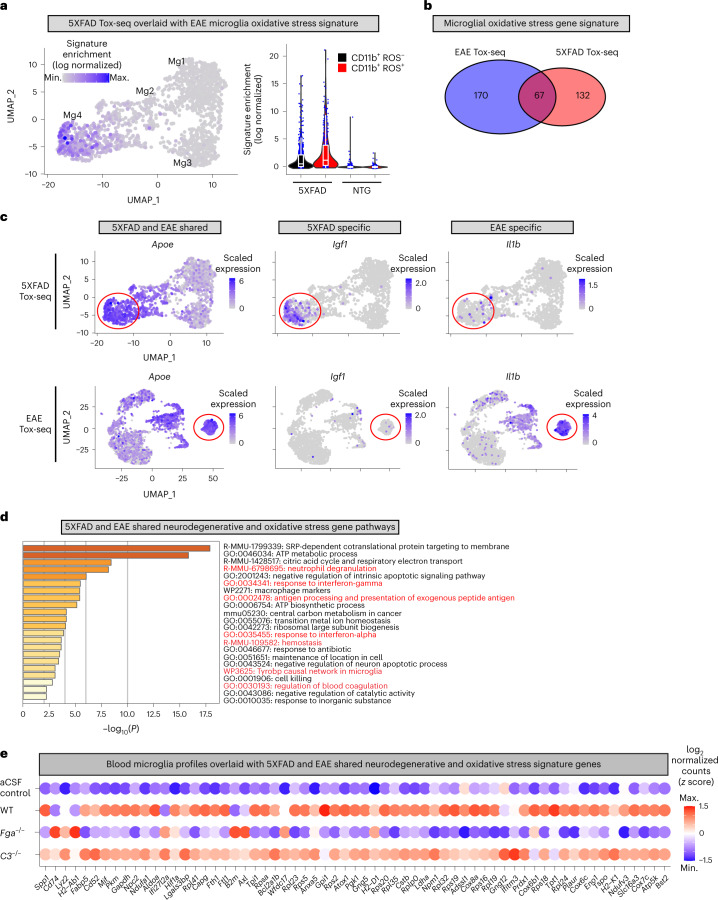
Comparison of microglial oxidative stress signature induced by neurodegeneration and autoimmunity. **a**, UMAP plot of 5XFAD Tox-seq clusters overlaid with EAE microglial oxidative stress genes signature (left). Violin plots of EAE oxidative stress signature enrichment in 5XFAD and NTG mice (right); *n* = 1,579 cells from brains of three 5XFAD and three NTG mice. Violin plots depict minimum, maximum and median expression, with points showing single-cell expression levels. Box plots show the first to third quartiles (25–75% box bounds) with median values indicated and upper and lower whiskers extending to 1.5× interquartile range. **b**, Venn diagram of oxidative stress genes in CD11b^+^ ROS^+^ microglia from 5XFAD or EAE. **c**, UMAP plots of shared and unique oxidative stress microglia genes in 5XFAD and EAE. Gene expression overlays for *Apoe*, *Igf1* and *Il1b* are shown. Gene expression is depicted as log-normalized scaled expression. The red outline demarcates microglial cells in a ROS-enriched cluster. Representative genes shared between or specific to 5XFAD and EAE Tox-seq are shown. **d**, Metascape analysis of top significant gene pathways shared in ROS^+^ microglia from 5XFAD and EAE mice. **e**, Dot plot of gene expression from blood microglia profiles overlaid with the 5XFAD and EAE shared oxidative stress gene signature in microglia as shown in **b**.

**Extended Data Fig. 10:**
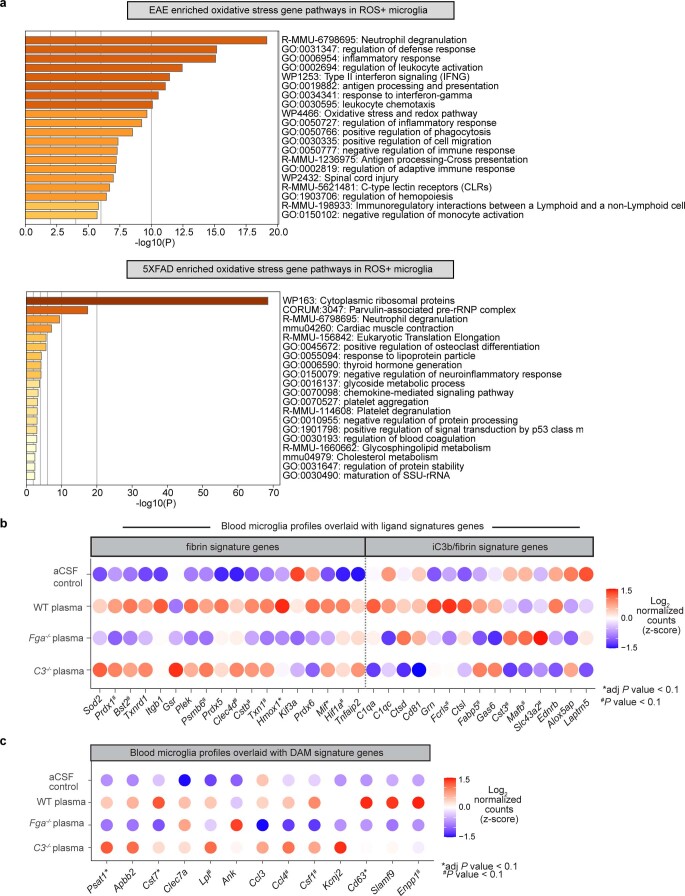
Pathway analysis of unique microglia oxidative stress gene signatures and gene signature overlays. **a**, Metascape analysis showing the top significant gene pathways shared in ROS+ microglia from EAE (top) and 5XFAD (bottom) mice. Data correspond to gene signatures in Fig. 8b. *P* values calculated by hypergeometric test without multiple test correction. **b, c**, Dot plot of microglia gene expression from plasma or control injected brains overlaid with microglia fibrin and iC3b/fibrin activation signature genes (**b**) or in vivo DAM signature (**c**). Data points represent the average of *n* = 3 - 4 biological replicates per group. Two mice were pooled per replicate. Significance determined by Wald test following by BH test correction.

We next compared the Tox-seq transcriptomic profiles of microglia between the 5XFAD and EAE models. The oxidative stress core signature identified in ROS^+^ microglia from EAE mice was also present in 5XFAD mice (Fig. 8a and Supplementary Table 16). Although 67 DEGs were shared between EAE and 5XFAD ROS^+^ microglia (for example, *Apoe*), the majority of genes were specific for either 5XFAD (132 DEGs) or EAE (170 DEGs), such as *Igf1* and *Il1b*, respectively (Fig. 8b,c). These results are in line with human microglial transcriptomics identifying partial overlap between MS and AD^37^. Pathway analysis of the microglial oxidative stress genes shared among EAE and 5XFAD models identified enrichment in pathways related to blood coagulation and hemostasis (for example, *Plaur*, *Slc16a3*, *Eno1*), antigen presentation (for example, *H2-Ab1*, *H2-K1*, *Cd74*), neutrophil degranulation (for example, *B2m*, *Cstb*, *Bst2*) and the tyrosine kinase binding protein Tyrobp network (Fig. 8d, Extended Data Fig. 10a and Supplementary Table 16). We next overlaid the shared AD and EAE oxidative stress signature with the blood-induced microglia profiles (Fig. 8e). The WT plasma signature overlapped with the shared oxidative stress microglia signature, indicating that the dataset alignment identified blood-induced microglia genes both in MS and AD mice. *Fga*^−*/*−^ plasma largely reduced oxidative stress and disease-associated transcripts to control levels (Fig. 8e and Extended Data Fig. 10b,c), suggesting that fibrinogen is a key ligand in the blood that activates neurodegenerative microglia responses. Taken together, these data suggest a pathogenic role for fibrin-induced microglia polarization in neurodegeneration in both MS and AD, demonstrating shared and unique drivers of innate immune-driven neurotoxicity.

## Discussion

We report the first unbiased transcriptome and phosphoproteome of blood-induced polarization of innate immunity, revealing the selectivity and causal role of blood proteins in mediating neurotoxic microglial functions. Traditionally, blood leaks were considered to be secondary to inflammation, with largely interchangeable functions once they extravasated into the brain^7,38^. Through in vivo genetic loss-of-function studies combined with unbiased comparative transcriptomics analysis, our study shows the specificity of blood proteins in differentially activating receptor-mediated immune responses required for pathogenic microglial gene programs in AD and MS models. Blood-induced prooxidant programming of neurotoxic microglia occurs along common molecular pathways across neurodegeneration and CNS autoimmunity. Fibrin–CD11b signaling was necessary for neurotoxic microglia programs in AD mice consisting of neurodegenerative, oxidative stress and lipid metabolism that were shared with an MS model. Given that immune, vascular and blood signals are key players in aging, neurological and peripheral diseases^2,4,39^, our dataset represents a valuable resource for the study of novel pathways and biomarkers and for discovery of drugs that selectively target pathogenic innate immunity in aging and inflammatory, autoimmune and neurodegenerative diseases.

Our study provides a mechanistic link between cerebral vascular pathology and neurodegeneration by identifying fibrin–CD11b signaling as an apical inducer of neurotoxic pathways in innate immune cells. Our model proposes that fibrin binding to CD11b–CD18 induces an outside-in integrin signaling cascade to initiate focal adhesion complex formation, activate MAPK and transactivate NADPH oxidase to induce proinflammatory, oxidative stress and IFN-I signaling responses. Fibrin also induces phosphorylation of SMARCA5, RANBP3 and NUP98, suggesting regulation of nuclear import, chromatin remodeling and transcription^40^. Formation of multiprotein adhesion complexes that link the ECM to the cytoskeleton, MAPK signaling, oxidative stress and gene expression identify fibrin as a mechanoregulator of CD11b–CD18 integrin effector functions in innate immune cells^27,41,42^. Indeed, increased fibrin deposition, paxillin, MAPK and sustained MEK2 activation, and NADPH oxidase activity promote oxidative injury and mediate neurodegeneration and synaptic dysfunction in both MS and AD^6,20,43–47^. In addition to NADPH oxidase, we identified mitochondrial VDAC1 and *ApoE* as induced by fibrin, suggesting that fibrin-induced oxidative stress may be mediated by multiple pathways. We also identified fibrin as a potent activator of STATs, ISGs and IFN-I response, which induces neuronal dysfunction in AD^48^ and T cell effector functions in MS and autoimmune diseases^49^. Fibrin could thus be a driver of IFN-I response in disease. Together, these results identify fibrin as a key signal required for pathogenic polarization of immune cells at sites of vascular damage.

We discovered unique and shared transcriptomic and phosphoproteomic signatures induced by fibrin and iC3b, indicating that ligand-biased CR3 signaling may underlie the pleiotropic functions of CR3 in innate immune cell polarization^50–52^. Our results suggest that limiting fibrin-induced innate immune responses may suppress oxidative injury and neurodegeneration, whereas suppressing complement may preferentially reduce phagocytosis and antiviral immune responses. iC3b-specific signatures could also be relevant to complement diseases such as C3 glomerulopathy^1^. Ligation of CR3 by fibrin and iC3b may lead to differential conformational changes in its ectodomain, leading to ligand-biased outside-in signaling^41^. The stoichiometry and spatial distribution of fibrin and iC3b may also contribute to biased ligand signaling and transactivation of other receptors. Fibrin deposits and complement activation may affect immunopathogenesis of intracerebral hematoma in conditions such as stroke and traumatic brain injury, in addition to neurodegenerative diseases^53^. Future studies will be necessary to determine how the cross-talk of the fibrinogen and complement pathways orchestrate oxidative injury and phagocytic signaling cascades, respectively. As CR3 mediates both protective and damaging immune functions, strategies to target ligand-selective activation pathways may have therapeutic benefits^8^. Fibrin–CD11b signaling is required for pathogenic innate immune activation in the brain and periphery^19,20,23,29,54–56^. *Fgg*^*γ390*–^^*396A*^ mice and those treated with the fibrin-targeting 5B8 antibody show protection against neurodegeneration and cognitive impairment in AD models and against paralysis and axonal damage in EAE^19–21,24^. Protection from neurodegeneration upon inhibition of fibrin–CD11b signaling may be due to selective suppression of neurotoxic pathways in microglia, such as ApoE, IFN-I and oxidative stress pathways identified in this study^32,34,49,57^. Thus, fibrin-targeting immunotherapy could be a therapeutic strategy in AD and MS without adverse anticoagulant effects or global suppression of innate immunity.

The resource we provide here should be considered with some caveats. As in vitro microglia cultures do not fully recapitulate in vivo homeostatic signatures^58^, we also validated the ligand-selective signatures in vivo. Antibody-depleted plasma for fibrin, C3 or other blood proteins could complement the genetic depletion used in this study. Our phosphoproteomic analysis was performed in RAW 264.7 cells owing to their transcriptomic similarity to BMDMs, their use in phosphoproteomic studies^25,59^ and because of the technical demand for high cell numbers. We validated fibrin-induced phosphosites identified in RAW 264.7 cells in BMDMs and primary microglia and in 5XFAD mice, suggesting that the phosphorylation events also occur in vivo. Future studies in primary cells with additional concentrations and time points could be used to assess differential signaling pathways. For our comparative transcriptomic analysis, we selected three ligands—fibrin, iC3b and LPS—in macrophages and microglia owing to their broad and diverse immune roles in vascular, inflammatory and infectious disease. We selected fibrin and iC3b to study ligand-biased signaling of CD11b–CD18. Future studies could use our platform to compare additional complement and coagulation activators in other cell types. Another potential limitation was that protein aggregates could induce signaling independent of specific ligand-receptor interactions. However, we found specific receptor-mediated transcriptomic programs for blood proteins in vivo and suppression of the neurodegenerative gene signature in 5XFAD*:Fgg*^*γ390*–*396A*^ mice, indicating the distinct transcriptional programs of blood proteins are not due to aggregate protein formation. Although the results of transcriptomic analysis in 5XFAD*:Fgg*^*γ390*–*396A*^ mice support the fibrin-induced microglia response being CD11b dependent, they do not exclude direct or indirect effects of fibrin on other cellular targets in the brain^3^. The Trem2 pathway was induced in microglia in 5XFAD mice but not in the healthy brain by the blood, potentially owing to the difference in pathology as well as the age of the mice and the time points in the study. We performed Tox-seq analysis in 5XFAD mice, an AD model dependent on immune and vascular mechanisms^20,60^. Future studies could use Tox-seq to characterize neurotoxic innate immune responses in other neurodegeneration models with different etiologies to discover additional pathways related to disease progression.

In summary, we demonstrated that blood-induced polarization of innate immunity is causal for the induction of neurotoxic microglial programming in disease. By establishing a blood-innate immunity multiomic and genetic loss-of-function pipeline, we defined fibrin as a unique blood protein required for microglial polarization to oxidative stress and neurodegenerative phenotypes in MS and AD mice. Our study uncovers principles of distinct transcriptomic and phosphoproteomic events induced by immune and vascular signals and their contributions to immune diversity in autoimmune and neurodegenerative disease. Furthermore, we lay the groundwork for future experiments to define the spatiotemporal regulation of blood-induced innate immune cell polarization, which may enable discovery of selective therapeutic strategies in inflammatory, neurological and infectious diseases.

## Methods

### Animals

C57BL/6J and B6.SJL-Tg (APPSwFlLon,PSEN1*M146L*L286V) 6799Vas/Mmjax (5XFAD) mice (034840-JAX) were purchased from the Jackson Laboratory (JAX) and backcrossed in a C57BL/6J background for more than 30 generations^61^. *Fga*^−/−^ and *Fgg*^γ390–396A^ mice^62,63^ were obtained from J. Degen (University of Cincinnati, OH, USA). 5XFAD mice were crossed with *Fgg*^*γ390*–^^*396A*^ mice to generate 5XFAD:*Fgg*^γ390–396A^ mice. Male and female mice were used in this study. Sprague–Dawley female rats with litters were purchased from Charles River. Animals were housed under Institutional Animal Care and Use Committee guidelines in a temperature and humidity-controlled facility with a 12 h light–12 h dark cycle and ad libitum feeding. Animal protocols were approved by the Committee of Animal Research at the University of California San Francisco and in accordance with the National Institutes of Health guidelines.

### Plasma injection for microglia RNA-seq

Citrate plasma from 8–11-week-old *C3*^−/−^ female (029661, JAX)^64^ and *Alb*^−/−^ female (025200, JAX)^65^ mice was obtained from JAX. Citrate plasma was isolated from WT C57BL/6J, *Fga*^−/−^ and *Fgg*^*γ390*–^^*396A*^ mice as previously described^21^. Plasma or artificial cerebrospinal fluid (aCSF) control was bilaterally injected (1.5 μl, 0.3 μl min^−^^1^) into the corpus callosum of male C57BL/6J mice, as previously described^21^. Twenty-four hours later, two mice were pooled per replicate, and microglia were isolated from 1-mm tissue blocks spanning the injection sites. Tissues were incubated in lysis buffer (1 mg ml^−1^ collagenase D (Sigma-Aldrich), 0.05 mg ml^−1^ DNase1 (Sigma-Aldrich), 3 μM actinomycin D (ActD) diluted in Dulbecco’s phosphate-buffered saline (DPBS) with Ca^2+^ Mg^2+^ (Thermo Fisher Scientific)) for 30 min at 37 °C. Myelin was depleted using a debris removal kit (Miltenyi Biotec) as previously described^66^. Cells were treated with Fc-block in fluorescence-activated cell sorting (FACS) buffer (DPBS supplemented with 0.2% bovine serum albumin, Thermo Fisher Scientific) for 5 min at 4 °C then incubated with primary antibodies for 30 min at 4 °C. The following primary antibodies from BioLegend were used at 1:200 dilution: CD11b (M1/70) and CD45 (30-F11). Then, 15,000 to 20,000 CD45^lo^ CD11b^+^ microglia were FACS sorted into tubes containing RLT plus lysis buffer (Qiagen) supplemented with 1% 2-mercaptoethanol and 0.25% reagent DX (Qiagen) using a FACSAria II with BD FACSDiva v.8 and FlowJo v.10 software for analyses. Cells were gated on side scatter area (SSC-A) and forward scatter area (FSC-A) size, and then doublet discrimination was performed with FSC-H and FSC-W (height and width) parameters. Microglia lysates were frozen and stored at −80 °C until they were processed for RNA-seq.

### Bulk RNA-seq of microglia

Samples from the plasma injection experiment were processed for RNA-seq using an Ovation RNA-seq System V2 low input kit (NuGEN) as previously described^31^. Libraries were equimolar pooled and sequenced on a NovaSeq 6000 S4 (Illumnia) with 200 paired-end reads to a depth of >50 million reads per library. Paired-end fastq files were processed using the Nextflow RNA-seq pipeline^67^ with nextflow v.20.12.0-edge in Singularity with default nf-core/rnaseq v.3.0 parameters and packages. Fastq files were mapped to the GRCm38/mm10 genome (downloaded from nf-core). Gene analyses were performed in R v.4.2.0 using DeSeq2 v.1.36.0 on the salmon.merged.gene_counts_scaled file. Reads with fewer than three counts per gene across replicates were filtered. Three samples did not pass RNA and cDNA library quality control (QC) testing; another was removed owing to large deviation on principal component analysis (PCA) and poor sequence alignment. No other samples or animals were excluded from analyses. For differential gene analysis, the *results* function in DeSeq2 was used with contrast to test between two genotypes and treatments of interest. Significance was determined by abs(log_2_ fold change (FC)) > 0.5 and adjusted *P* < 0.1 unless otherwise stated. Unbiased KEGG analysis was performed using clusterProfiler with default parameters and pvalueCutoff set to 0.1. The blood microglia gene network was generated in Cytoscape v.3.7.2 (ref. ^68^) using upregulated DEGs identified in WT plasma compared with aCSF samples (Supplementary Table 3). GO pathways were determined using functional enrichment analysis in String^69^ with default parameters visualized in Cytoscape.

### Cell culture

Primary microglia were prepared from neonatal rats at postnatal day 5 or from C57BL/6J mice at postnatal days 2–3 as previously described^21,31^. Viability was assessed using trypan blue. Microglia cultures from one litter were used for each independent experiment. BMDMs were prepared from male and female 12–20-week-old mice as previously described^21,31^ and used for experiments after 6 or 7 days of differentiation. For BMDM scRNA-seq, two male or two female mice were pooled per experiment. Individual animals were used for biological replicates unless otherwise stated. RAW 264.7 macrophages were obtained from ATCC and cultured in Dulbecco’s modified Eagle medium (DMEM) supplemented with 10% fetal bovine serum (FBS).

### scRNA-seq of ligand stimulated cells

Fibrin-coated 24-well culture plates (Corning) were prepared as previously described^29^. Then, 10 μg ml^−1^ iC3b (CompTech; A115) in HEPES pH 7.2 buffer was immobilized on culture plates by 1 h incubation at 37 °C followed by overnight incubation in a 37 °C humidified chamber. For fibrin or iC3b stimulation, BMDMs or primary microglia were seeded into fibrin-coated or iC3b-coated wells at 5.0 × 10^5^ cells ml^−1^. Control wells were treated with the same buffer void of fibrin or iC3b. For LPS stimulation, BMDMs or microglia were seeded into 24-well tissue-culture-treated plates at 5.0 × 10^5^ cells ml^−1^, allowed to adhere overnight, then treated with 100 ng ml^−1^ LPS (Sigma-Aldrich, O55:B5) for the durations indicated in figure legends. Following stimulation, adherent cells were lifted with accutase (StemCell Technologies) and viable cells were counted by trypan blue, then resuspended in RPMI with 5% FBS and used for scRNA-seq.

For scRNA-seq library preparation, BMDMs or primary microglia were processed with a Chromium Single Cell 3′ v.2 kit according to the manufacturer’s guidelines (10x Genomics). Libraries were balanced to achieve a minimum of 75,000 reads per cell and run on two lanes of a NovaSeq 6000 (Illumnia) with 150 paired-end reads. Samples were demultiplexed, and fastq files were used to align reads to the mm10 reference assembly (downloaded 2019) and aggregated using the Cell Ranger count and aggr packages (10x Genomics).

### 5XFAD Tox-seq

Samples were prepared for Tox-seq analysis as previously described^31^ with the following modifications. Male and female 12-month-old 5XFAD and NTG mice were perfused with 4 °C DPBS, and cortical and hippocampal regions were dissected. Tissues were incubated with lysis buffer without ActD for 30 min at 37 °C. Using a FACSAria II, live Sytox blue^−^ CD11b^+^ ROS^−^ and live Sytox blue^−^ CD11b^+^ ROS^+^ cell populations were sorted into tubes containing FACS buffer. Sorted cells were resuspended in 4 °C DPBS supplemented with 2% FBS and immediately processed for scRNA-seq.

For scRNA-seq library preparation, live Sytox blue^−^ CD11b^+^ ROS^−^ and live Sytox blue^−^ CD11b^+^ ROS^+^ sorted cell populations were processed using the Chromium Single Cell 3′ v.2 kit following the manufacturer’s instructions. Balanced library pools were sequenced across three lanes of a HiSeq 4000 system (Illumnia) with a targeted sequencing depth of 100,000 reads per cell. Reads were mapped to the mm10 genome (downloaded 2019), and samples were combined and sequence-depth normalized using Cell Ranger count v.3.0.2 and aggr packages, respectively.

### Brain CD11b scRNA-seq

Brains from male and female 6-month-old 5XFAD, 5XFAD:*Fgg*^*γ390*–^^*396A*^, NTG and *Fgg*^*γ390*–^^*396A*^ mice were processed for FACS as described for 5XFAD Tox-seq analysis. Live Sytox blue^–^ CD11b^+^ cells from cortical/hippocampal tissues were sorted into tubes containing DPBS supplemented with 5% FBS at 4 °C and then resuspended in 4 °C DPBS supplemented with 2% FBS at 333 cells μl^−1^ and processed for scRNA-seq with the Chromium Single Cell 3′ v.3 kit following manufacturer’s instructions. Balanced library pools were run across three lanes of Hiseq4000, reads mapped to the mm10 genome and samples combined and sequence-depth normalized using the Cell Ranger count v.3.0 and aggr packages, respectively.

### scRNA-seq data analysis

The R toolkit Seurat^70^ was used for QC, clustering analysis and differential gene expression analysis of scRNA-seq data in R v.4.0.2 unless otherwise stated. For scRNA-seq data visualizations, dittoseq package v.1 was used to produce UMAPs, dot plots and violin plots^71^.

For microglia scRNA-seq analysis (Fig. 2), the QC parameters were: nFeature_RNA > 1000; nFeature_RNA < 5500; and < 5% and 20% mitochondrial and ribosomal genes, respectively. nCount_RNA in the 93rd percentile (nCount_RNA < 26,206) was used for downstream analysis. Data were normalized and scaled, and a percentage of mitochondrial and cell cycle genes were regressed out using Seurat SCTransform. Jackstraw was performed with num.replicate of 100. The FindNeighbors and FindClusters functions in Seurat were used with the first eight significant principal components (PCs) and a resolution of 0.4, respectively. A total of 16,186 microglial cells passed QC, with an average of 3,469 genes per cell and 20,228 genes. Consistent with the literature^72^, canonical microglial markers were expressed at varying levels in the identified clusters (Extended Data Fig. 3e). Cluster DEGs were determined by FindAllMarkers with default parameters. Genes that met the log_2_ FC threshold of >0.25 with adjusted *P* < 0.05 (Benjamini–Hochberg correction) were used for downstream analysis.

For BMDM scRNA-seq analysis (Fig. 3), two independent experiments were integrated and corrected for batch effects as previously described^70^. The batch-corrected dataset QC parameters were: nFeature_RNA > 200; nFeature_RNA < 5000; and < 5% and 25% mitochondrial and ribosomal genes, respectively. The 17,625 QC-passed BMDMs were used with the Seurat integration workflow using default parameters. Jackstraw was performed with num.replicate of 100. The RunUMAP, FindNeighbors and FindClusters functions were used with the first 20 significant PCs and a resolution of 0.5. DEGs were determined by FindAllMarkers with default parameters. Genes that met the log_2_ FC threshold of 0.25 with adjusted *P* < 0.05 (Benjamini–Hochberg correction) were used for downstream analysis. Pseudotime trajectories were performed on the UMAP embeddings and Seurat clusters using Slingshot v.2.2.1 (ref. ^73^), where cluster 3 was the predefined start point. Associations between gene expression pattern and pseudotime were tested for each lineage by fitting a negative binomial generalized additive model at 8 knots using tradeSeq v.1.8.0 (ref.^74^). The estimated smoothers for each lineage accounted for batch effects. For each lineage, markers differentially expressed between the average of the start and end points of a trajectory were identified. Adjusted *P* values (Benjamini–Hochberg correction) were used to identify the top 50 genes for each lineage (Supplementary Table 6). To generate the heat map, a pseudocount of 1 was added to the raw scRNA-seq counts for the top 50 DEGs, the rows were log_2_ row-normalized and *K*-means clustering was performed on the rows.

For 5XFAD Tox-seq analysis (Fig. 6), the QC parameters were 200–5,000 nFeature_RNA, <7,500 nCount_RNA, and <5% and 20% mitochondrial and ribosomal genes, respectively. Data were normalized and scaled, and a percentage of mitochondrial genes were regressed out using Seurat SCTransform. Jackstraw was performed with num.replicate of 100. FindNeighbors and FindClusters Seurat functions were used with the first 30 significant PCs and a resolution of 0.6, respectively. To remove variation in sex-linked genes, the dataset was integrated using the Harmony algorithm^75^ with runHarmony: group.by.vars = sex and assay.use = SCT. Clustering analysis was performed using Harmony dims = 15 and resolution = 0.4. In accordance with previous literature^76^, all four CD11b^+^ cell clusters had high expression of core microglial genes (Extended Data Fig. 9e and Supplementary Table 13). DEGs for each cluster were determined by FindAllMarkers with default parameters using MAST statistical test. Genes that met the log_2_ FC threshold of 0.25 with adjusted *P* < 0.05 (Benjamini–Hochberg correction) were used for downstream analyses.

For brain CD11b^+^ scRNA-seq analysis (Fig. 7), the QC parameters were: 1,000–4,000 nFeature_RNA, <12,000 nCount_RNA, <10% mitochondrial genes. Batch correction was performed using FindIntegrationAnchors for ‘batch1’ and ‘batch2’. The ScaleData function was performed and microglia immediate response genes were regressed out using the vars.to.regress function set to c(‘Fos’,‘Egr1’,‘Jun’,‘Junb’,‘Zfp36’,‘Jund’,‘H3f3b’, ‘Btg2’,‘Rhob’,‘Fosb’,‘Dusp1’,‘Ier2’,‘Socs3’,‘Ier5’,‘Nfkbia’,‘Zfp36l1’,‘Btg1’,‘Ptma’,‘Sgk1’,‘Klf6’). FindNeighbors and FindClusters were used with the first 20 significant PCs and a 0.2 resolution, respectively. Differential gene analysis was performed using FindMarkers or FindAllMarkers with MAST or Wilcoxon test for p_val_adj < 0.05 and avg_log2FC > 0.25.

### scRNA-seq signature enrichment

Average expression levels for a given gene list were computed across single-cell transcriptomes using the AddModuleScore function in Seurat with default parameters. The modular scores of a gene list were visualized in UMAP or violin plot. The list of genes used is provided in Supplementary Table 12.

### Functional enrichment and network analysis of scRNA-seq data

Functional enrichment analysis of DEGs was performed in Metascape using default parameters^77^, and significant GO terms were identified by false discovery rate (FDR) *P* < 0.05 unless otherwise stated. Gene network analyses were performed with GSEA with C5.bp.v7.1symbols.gmt using default settings. GO terms with *P* < 0.10 were used for enrichment map visualization in Cytoscape v.3.7.2 and unbiasedly clustered using the AutoAnnotate v.1.3.2 plugin with default settings. For the microglial dataset, cluster gene signatures were determined using ClusterProfiler^78^ and the gseGO function with the following parameters: ont = BP, nPerm = 10000, minGSSize = 3, maxGSSize = 800, pvalueCutoff = 0.1, OrgDB = org.Mm.eg.db, pAdjustMethod = BH.

### Phosphoproteomics sample preparation

RAW 264.7 macrophages (10 × 10^6^ cells, 10 mg of protein per sample) were prepared for global phosphorylation protein sample digestion for mass spectrometry analysis as previously described^79^. Macrophages were plated on fibrin-coated (final concentration, 12.5 μg ml^−1^) or iC3b-coated (final concentration, 10 μg ml^−1^) plates for 1 or 3 h. Fibrin concentration was based on our previous studies^21,29,31^. We selected a comparable concentration for iC3b based on previous studies using similar concentrations for macrophage effector responses and CD11b binding^80^. Under these conditions, in our previous studies, we observed phosphorylation at longer time points in primary Schwann cells^81^. RAW cells were used owing to the high protein concentration needed for phosphoproteomic analysis, which could not be feasibly obtained from primary BMDM cultures using instrumentation at the time of the study.

### Mass spectrometry analysis

Samples were analyzed on a Thermo Scientific Orbitrap Fusion mass spectrometry system equipped with an Easy nLC 1200 uHPLC system interfaced with the mass spectrometer via a Nanoflex II nanoelectrospray source as previously described^79^.

### Mass spectrometry data processing and statistical analysis

Quantitative analysis was performed in R v.4.1.3. Initial QC analyses, including interrun clusterings, correlations, PCA, and peptide and protein counts and intensities were completed with the R package artMS v.1.12.0. Two sample outliers in intensities and peptide detections were discarded before quantitative analysis: fibrin 1 h (PRIDE sample ID FU20180420-23) and iC3b 1 h (PRIDE sample ID FU20180420-05). Statistical analyses of phosphorylation changes between stimulated and control runs were carried out using peptide ion fragment intensity data output from MaxQuant with preprocessing using artMS. Quantifications of phosphorylation based on peptide ions were performed with artMS::doSiteConversion and artMS::artmsQuantification with default settings using artMS^82^. All peptides containing the same set of phosphorylated sites were grouped and quantified together into phosphorylation site groups, and equal median normalization was performed across runs to control for differences in sample preparation. Statistical tests in MSstats compared phosphopeptide intensities between stimulated and control conditions for each time point. We compared each stimulation condition with its time-matched control and compared stimulations with each other (that is, fibrin versus iC3b). We used defaults for MSstats for adjusted *P* values (Student’s *t* test and Benjamini–Hochberg correction), even in cases of *n* = 2 biological replicates. We quantified between 2,000 and 6,000 phosphorylated peptides per sample, mapping to 300–3,000 different proteins per sample.

### Kinase activity analysis

FC values from MSstats were reduced to a single FC per site by choosing the FC with the lowest *P* value (noninfinite log_2_-transformed FC values) and used for kinase activity and enrichment analysis. *Mus musculus* phosphorylation sites were converted to their *Homo sapiens* orthologous sites. Orthologous pairs of gene identifiers between *M. musculus* and *H. sapiens* were downloaded from Ensembl using BioMart. Ensembl gene identifiers were mapped to UniProt identifiers, and orthologous pairs of sequences were aligned using the Needleman–Wunsch global alignment algorithm implemented using the Biostrings v.2.62.0 function pairwiseAlignment with default parameters in R. The resulting alignments were used to convert the sequence positions of phosphorylations in *M. musculus* to positions in *H. sapiens* protein sequences, if possible. Kinase activities were estimated using known kinase–substrate relationships^83^ and inferred as a *z* score calculated using the mean log_2_ FC of phosphorylated substrates for each kinase in terms of standard error (*z* = (*M* − *u*)/s.e.), comparing FCs in phosphosite measurements of the known substrates against the overall distribution of FCs across the sample. *P* values were calculated using two-tailed *z* test^84^. We collected substrate annotations for 400 kinases with available data. Kinases with two or more measured substrates were considered to be predicted kinases (Supplementary Table 11).

### Network reconstruction and enrichment analysis of phosphoproteomics data

Proteins with changes in phosphorylation state were selected based on an FDR threshold of 0.05. Protein phosphorylation site pairs significant for at least one time point were maintained. After filtering, iC3b resulted in 44 phosphoproteins, and fibrin resulted in 68 phosphoproteins. The STRING database was queried using Cytoscape. Proteins with STRING interaction scores higher than 0.4 were connected by edges with widths and opacities reflecting the score level. Phosphorylation state changes were visualized using Omics Visualizer^85^ as two outer ring circles representing phosphorylation at 1 h and 3 h. To enhance the signal, we included up to ten additional nodes identified by the STRING database as functionally related to our phosphoproteins using stringApp^86^. Final results were filtered based on an FDR threshold of 0.05, and redundant results were removed using a redundancy cutoff of 0.5. Two significant GO terms were selected and visualized as node fill colors. STRING-provided proteins and unconnected proteins were removed for visualization.

### Fibrin phosphorylation cell assays

BMDMs were cultured for 18 h in RPMI-1640 supplemented with 1% FBS (RPMI 1% FBS). Cells were plated on fibrin-coated dishes for 15-90 min in RPMI 1% FBS and then processed for either immunocytochemistry (ICC) or immunoblotting. Unstimulated BMDMs served as controls. Primary rat microglia were used on day 4 in vitro and plated on fibrin-coated dishes for 15–75 min in DMEM supplemented with 2% FBS. Unstimulated microglia served as controls.

### Pharmacologic inhibition assays

BMDMs were cultured for 18 h in RPMI-1640 supplemented with 1% serum. For MEK inhibition, cells were preincubated with 20 nM trametinib (S2673, Selleckchem) for 2 h and then plated on fibrin-coated plates for 90 min for ICC or 6 h for quantitative PCR. Cells unstimulated in RPMI 1% FBS for 90 min or 6 h were used as time point zero controls. Dimethyl sulfoxide was used as a vehicle control. Fibrin–CD11b blockade using 5B8 monoclonal antibody was performed as previously described^29^. In brief, 5B8 or IgG2b isotype control antibodies were preincubated (each 50 μg ml^−1^) in fibrin-coated plates for 90 min at 37 °C before cell plating. Cells were incubated on fibrin for 90 min and processed for ICC. Cells incubated with 5B8 or IgG2b in the absence of fibrin served as controls.

### Immunohistochemistry and Immunocytochemistry

Brains were processed for immunohistochemistry as described^29,31^. The following antibodies were used: mouse anti-GP91phox (1:150; 53, BD Biosciences), rabbit anti-IBA1 (1:500; 019-19741, Wako), goat anti-APOE (1:50; AB947, MilliporeSigma), and Alexa 647, 488 and Cy3 (1:500; Jackson ImmunoResearch). Amyloid plaques were labeled with 5 mg/mL Methoxy-X04 (Tocris) for 30 min at 23 °C. Confocal images were acquired with a Fluoview FV1000 (Olympus) confocal microscope and Fluoview software v.3.1b with Olympus x40 and 0.8 NA water-immersion lens. Images of the CA3 hippocampus and periplaque were quantified using NIH ImageJ (v.1.50). Image acquisition and quantification was performed by observers blinded to experimental conditions.

ICC was performed as previously described^87^ with the following modifications. Briefly, BMDMs were seeded into 16-well chamber glass slides (Nunc) coated with fibrin for various times in a 5% CO_2_ incubator at 37 °C. Cells were allowed to adhere for 15–30 min before time point collection. Cells unstimulated in RPMI 1% FBS overnight were used as time point zero controls unless otherwise stated. Primary antibodies for p-NCF2 (1:500, rabbit polyclonal, PA5-105094, Thermo Fisher Scientific), p-PXN (1:500, rabbit polyclonal, PAB7932, Abnova) and p-MEK2 (1:500, rabbit polyclonal, 28955-1-AP, Thermo Fisher Scientific) were incubated overnight at 4 °C. Actin was stained with Alex Fluor Plus 555 Phalloidin, and nuclei were visualized with DAPI following the manufacturer’s instructions (Thermo Fisher Scientific). Images were acquired with an Axioplan II epifluoresence microscope (Zeiss) as previously described^31^, or Z stack images were taken with an LSM880 confocal microscope (Zeiss) using a ×63 objective. Image quantification was performed as previously described^66,87^. In ImageJ, a cell mask was created using phalloidin staining, then immunoreactivity was measured within the mask using the same threshold across images and presented as a percentage area.

### Immunoblots

Immunoblot analysis was performed as previously described^29^ with the following sample preparation specification. Primary microglia were washed in 4 °C DPBS and then incubated in Pierce RIPA lysis buffer supplemented with 1× Halt Protease and phosphatase inhibitors (complete RIPA; Thermo Fisher Scientific) for 15 min at 4 °C. For brain samples, cortices were dissected from PBS-perfused 12-month-old male and female mice. Tissues were incubated in complete RIPA for 30 min at 4 °C. Primary antibodies were: p-NCF2 (1:1000, rabbit polyclonal, PA5-105094, Thermo Fisher Scientific); NCF2 (1:1000, rabbit polyclonal, PA5-37323, Thermo Fisher Scientific), p-PXN (1:1000, rabbit polyclonal, PAB7932, Abnova), paxillin (1:1000, rabbit monoclonal, ab32115, Abcam), p-MEK2 (1:1000, rabbit polyclonal, 28955-1-AP, Thermo Fisher Scientific), MEK1/2 (1:10,000, rabbit monoclonal, ab178876, Abcam) and GAPDH (1:10,000, rabbit monoclonal, 2118, Cell Signaling Technology). Primary antibodies were visualized with horseradish peroxidase-conjugated secondaries (Cell Signaling Technology) and ECL reagents. Densitometry was performed using NIH ImageJ (v.1.50), with protein values for each band normalized to GAPDH from the same membrane.

### Quantitative real-time PCR

Quantitative PCR and data analysis were performed as previously described^31^. The following primer sequences were used. *Gapdh*: forward, caaggccgagaatgggaag; and reverse, ggcctcaccccatttgatgt. *Il1b*: forward, agttgacggaccccaaaag; and reverse, agctggatgctctcatcagg.

### Statistical analyses for nonsequencing data

Data are presented as mean ± s.e.m. with overlaid scatter plot. Data distribution was assumed to be normal, but this was not formally tested. Two-tailed unpaired *t* test or Mann–Whitney test, one-way analysis of variance (ANOVA) and two-way ANOVA tests were performed with GraphPad Prism v.9. No statistical method was used to predetermine sample sizes, but sample sizes were similar to those used in our previously published studies^29,31^. All mice survived until the end of the study, and all of the data were analyzed. Mice were randomized and blindly coded for group assignment and data collection for immunohistochemistry and ICC experiments. For in vivo stereotactic plasma injections, mice were randomized and blindly coded for group assignment and data collection. For all scRNA-seq experiments, mice were randomized by sex and genotype before sample preparation. All injections, histological analyses and quantification were done in a blinded fashion. Quantification of immunohistochemistry data was performed independently by two blinded observers.

### Reporting summary

Further information on research design is available in the Nature Portfolio Reporting Summary linked to this article.

## Online content

Any methods, additional references, Nature Portfolio reporting summaries, source data, extended data, supplementary information, acknowledgements, peer review information; details of author contributions and competing interests; and statements of data and code availability are available at 10.1038/s41590-023-01522-0.

## Supplementary information

Reporting Summary

Supplementary Tables Supplementary Tables 1–16 and table legends.

## Data Availability

The scRNA-seq and bulk RNA-seq datasets are deposited in the Genome Expression Omnibus under SuperSeries accession number GSE229376. Searchable web resources from this study of the microglia and BMDM ligand-activation scRNA-seq data are available at https://toxseq.shinyapps.io/ligand_activation/, and the single-cell 5XFAD Tox-seq data are available at https://toxseq.shinyapps.io/5xfad_toxseq/. The EAE Tox-seq data are available at https://toxseq.shinyapps.io/scrnaseq_viewer/. The mass spectrometry proteomics data have been deposited to the ProteomeXchange Consortium via the PRIDE partner repository with the dataset identifier PXD021230. The phosphoproteomic interaction networks have been made available through NDEx at 10.18119/N9F317 (fibrin network), 10.18119/N91S5X (iC3b network), 10.18119/N95K6G (fibrin GO-enriched subnetwork) and 10.18119/N9990P (iC3b GO-enriched subnetwork). Source data are provided with this paper.

## Source data

Source Data Fig. 4 Statistical source data.
Source Data Fig. 6 Statistical source data.
Source Data Fig. 7 Statistical source data.
Source Data Fig. 4 Unprocessed immunoblots.
Source Data Extended Data Fig. 6 Statistical source data.
Source Data Extended Data Fig. 8 Statistical source data.
