# Supplementary material for: Defining blood-induced microglia functions in neurodegeneration through multiomic profiling
Source: Nat Immunol. 2023 Jun 8;24(7):1173–87. doi: 10.1038/s41590-023-01522-0 (PMC10307624; doi:10.1038/s41590-023-01522-0)
Supplement: Supplementary file 1 — Reporting Summary [file 41590_2023_1522_MOESM1_ESM.pdf]

nature portfolio

Corresponding author(s): Katerina Akassoglou, PhD

Last updated by author(s): Apr 18, 2023

Reporting Summary

Nature Portfolio wishes to improve the reproducibility of the work that we publish. This form provides structure for consistency and transparency in reporting. For further information on Nature Portfolio policies, see our Editorial Policies and the Editorial Policy Checklist.

Statistics

For all statistical analyses, confirm that the following items are present in the figure legend, table legend, main text, or Methods section.

n/a	Confirmed	
☐	☒	The exact sample size (*n*) for each experimental group/condition, given as a discrete number and unit of measurement
☐	☒	A statement on whether measurements were taken from distinct samples or whether the same sample was measured repeatedly
☐	☒	The statistical test(s) used AND whether they are one- or two-sided<br>*Only common tests should be described solely by name; describe more complex techniques in the Methods section.*
☒	☐	A description of all covariates tested
☐	☒	A description of any assumptions or corrections, such as tests of normality and adjustment for multiple comparisons
☐	☒	A full description of the statistical parameters including central tendency (e.g. means) or other basic estimates (e.g. regression coefficient) AND variation (e.g. standard deviation) or associated estimates of uncertainty (e.g. confidence intervals)
☐	☒	For null hypothesis testing, the test statistic (e.g. *F*, *t*, *r*) with confidence intervals, effect sizes, degrees of freedom and *P* value noted<br>*Give P values as exact values whenever suitable.*
☒	☐	For Bayesian analysis, information on the choice of priors and Markov chain Monte Carlo settings
☒	☐	For hierarchical and complex designs, identification of the appropriate level for tests and full reporting of outcomes
☒	☐	Estimates of effect sizes (e.g. Cohen's *d*, Pearson's *r*), indicating how they were calculated

Our web collection on statistics for biologists contains articles on many of the points above.

Software and code

Policy information about availability of computer code

Data collection

Single cell transcriptomes were prepared using 10X Genomics Chromium Controller and sequenced using Illumnia Novaseq6000 and Hiseq4000. Cells were sorted using BD Biosciences FACSAria II with BD FACSDiva v8 and analyzed with FlowJo software v10. Proteomics was performed on a thermo Scientific Orbitrap Fusion mass spectrometry system equipped with an Easy nLC 1200 uHPLC system interfaced with the mass spectrometer via a Nanoflex II nanoelectrospray source. BD FACSDiva v8 and FlowJo software v10 were used.

Data analysis

Bulk RNA-seq of microglia. To facilitate reproducible RNA-seq data analysis, samples were processed using the open-access Nextflow RNA-seq pipeline59 using nextflow v20.12.0-edge in Singularity with default nf-core/rnaseq v3.0 parameters for paired-end FASTQ files. The following packages were used: Bioconductor-summarized experiment v1.20.0; bedtools v2.29.2; deseq2 v 1.28.0; dupradar v1.18.0; fastqc v0.11.9; picard v2.23.9; preseq v2.0.3; rseqc v3.0.1; salmon v.1.4.0; samtools v1.10; star v2.6.1d; stringtie v2.1.4; subread 2.0.1, trimgalore v0.6.6 and ucsc v377. FASTQ files were mapped to GRCm38/mm10 genome (downloaded from nf-core). Gene analyses was performed in R v4.2.0 using DeSeq2 v1.36.0 on the salmon.merged.gene_counts_scaled file produced by Nextflow. Reads with fewer than 3 counts per gene across replicates were filtered out. One outlier sample from groups Fga–/–, WT, Fggγ390-396A and aCSF were removed from downstream analyses due to large deviation on PCA and poor sequence alignment. For differential gene analysis the results function in DeSeq2 was used with contrast to test between two genotypes and treatments of interest. The results were then filtered for significance using log2foldchange > 0.5 and padj < 0.1 unless otherwise stated. KEGG analysis was performed using clusterProfiler v4.4.4 with default parameters and pvalueCutoff set to 0.1. The blood microglia gene network was generated and visualized in Cytoscape v3.7.260 using upregulated DEGs identified between WT plasma compared to aCSF samples (Supplementary Table 3). GO pathways were determined using functional enrichment analysis in String package61 with default parameters visualized in Cytoscape.

scRNA-seq data analysis. The R toolkit Seurat was used for QC, clustering analysis, and differential gene expression analysis of scRNA-seq data and performed in R v.4.0.2 unless otherwise stated. For scRNA-seq data visualizations, dittoseq package v1 was used to produce UMAPs, dot plots, and violin plots73.
For microglia scRNA-seq analysis (Fig. 2), QC parameters were: nFeature_RNA > 1000; nFeature_RNA < 5500; < 5% and 20% mitochondrial and ribosomal genes, respectively. nCount_RNA in the 93rd percentile (nCount_RNA < 26,206) were used for downstream analysis. Data were

normalized, scaled, and percent of mitochondrial and cell cycle genes regressed out following Seurat SCTransform. Jackstraw was performed with num.replicate of 100. FindNeighbors and FindClusters functions in Seurat were used with the first 8 significant PCs and a resolution of 0.4 resolution, respectively. 16,186 microglial cells passed QC with an average of 3,469 genes per cell with 20,228 genes. Consistent with literature, canonical microglial markers were expressed at varying levels in the identified clusters (Extended Data Fig. 3e). Cluster DEGs were determined by FindAllMarkers with default parameters. Genes that met log2fc.threshold > 0.25 with adjusted P value < 0.05 (Benjamini-Hochberg correction) were used for downstream analysis.
For BMDM scRNA-seq analysis (Fig. 3), two independent experiments were integrated and corrected for batch effect as described. Batch-corrected dataset QC parameters were: nFeature_RNA > 200; nFeature_RNA < 5000; < 5% and 25% mitochondrial and ribosomal genes, respectively. 17,625 QC-passed BMDMs were used with Seurat integration workflow using default parameters. Jackstraw was performed with num.replicate of 100. RunUMAP, FindNeighbors and FindClusters functions were used with the first 20 significant PCs and a resolution of 0.5. DEGs were determined by FindAllMarkers with default parameters. Genes that met log2fc.threshold,0.25 with adjusted P value < 0.05 (Benjamini-Hochberg correction) were used for downstream analysis. Pseudotime trajectories were performed on the UMAP embeddings and Seurat clusters using Slingshot v2.2.175, where cluster 3 was the predefined start point. Associations between gene expression pattern and pseudotime were tested for each lineage by fitting a negative binomial generalized additive model at 8 knots using tradeSeq v1.8.076. The estimated smoothers for each lineage accounted for batch effects. For each lineage, markers differentially expressed between the average of the start and end points of a trajectory were identified. Adjusted P value (Benjamini-Hochberg correction) was used to identify the top 50 genes for each lineage (Supplementary Table 6). To generate the heatmap, a pseudocount of 1 was added to the raw single cell RNA-seq counts for the top 50 DEGs, the rows were log2 row-normalized and K-means clustering was performed on the rows.
For the 5XFAD Tox-seq analysis (Fig. 6), QC parameters were 200 – 5,000 nFeature_RNA, < 7,500 nCount_RNA, < 5% and 20% mitochondrial and ribosomal genes, respectively. Data were normalized, scaled and percent of mitochondrial genes regressed out following Seurat SCTransform. Jackstraw was performed with num.replicate of 100. FindNeighbors and FindClusters Seurat functions were used with the first 30 significant PCs and a resolution of 0.6 resolution, respectively. To remove variation in sex-linked genes, the dataset was integrated with Harmony algorithm with runHarmony: group.by.vars = sex and assay.use = SCT. Clustering analysis was performed using Harmony dims = 15 and resolution = 0.4. In accordance with prior literature78, all four CD11b+ cell clusters had high expression of core microglial genes (Extended Data Fig. 8e and Supplementary Table 13). DEGs for each cluster were determined by FindAllMarkers with default parameters using MAST statistical test. Genes that met log2fc.threshold,0.25 with adjusted P value < 0.05 (Benjamini-Hochberg correction) were used for downstream analyses.
For brain CD11b+ scRNA-seq analysis (Fig. 7), QC parameters were: 1,000 – 4,000 nFeature_RNA, < 12,000 nCount_RNA, < 10% mitochondrial genes. Batch correction was performed using FindIntegrationAnchors for “batch1” and “batch2”, ScaleData function was performed on integrated object regression out known microglia immediate response genes. The vars.to.regress function was set to c(‘Fos’,‘Egr1’,‘Jun’,‘Junb’,‘Zfp36’,‘Jund’,‘H3f3b’, ‘Btg2’,‘Rhob’,‘Fosb’,‘Dusp1’,‘Ier2’,‘Socs3’,‘Ier5’,‘Nfkbia’,‘Zfp36l1’,‘Btg1’,‘Ptma’,‘Sgk1’,‘Klf6’). FindNeighbors and FindClusters were used with the first 20 significant PCs and a 0.2 resolution, respectively. Differential gene analysis was performed using FindMarkers or FindAllMarkers with MAST or Wilcoxon test for p_val_adj < 0.05 and avg_log2FC > 0.25.

scRNA-seq signature enrichment. The average expression levels of a given gene list was computed across single-cell transcriptomes using the AddModuleScore function in Seurat with default parameters. The modular scores of a gene list (i.e., signature enrichment) were visualized in UMAP or violin plot. The list of genes used are provided in Supplementary Table 12.

Functional enrichment and network analysis of scRNA-seq data. For in vitro datasets, functional enrichment analysis of DEGs was performed in Metascape using default parameters69, and significant GO terms were identified by FDR P value < 0.05 unless otherwise stated. Gene network analyses were performed with GSEA with molecular signatures database biological process for GO (C5.bp.v7.1symbols.gmt) using default settings. GO terms with P value < 0.10 were used for Enrichment Map Visualization in Cytoscape v.3.7.2 and were unbiasedly clustered using the plugin AutoAnnotate v. 1.3.2 with default settings. For the microglial dataset, cluster gene signatures were determined using ClusterProfiler v4.4.4 and gseGO function with the following parameters: ont = BP, nPerm = 10000, minGSSize = 3, maxGSSize = 800, pvalueCutoff = 0.1, OrgDB = org.Mm.eg.db, pAdjustMethod = BH.

Mass spectrometry data processing and statistical analysis. Quantitative analysis was performed in the R v.4.1.3. Initial quality control analyses, including inter-run clusterings, correlations, principal component analysis (PCA), peptide and protein counts and intensities were completed with the R package artMS v1.12.0. Two sample outliers in intensities and peptide detections were discarded prior to quantitative analysis: Fibrin 1 h (PRIDE sample ID FU20180420-23) and one iC3b 1 h (PRIDE sample ID FU20180420-05) samples. Statistical analysis of phosphorylation changes between stimulated and control runs were computed using peptide ion fragment intensity data output from MaxQuant and pre-processed using artMS. Quantifications of phosphorylation based on peptide ions were processed using artMS as a wrapper around MSstats86, via functions artMS::doSiteConversion and artMS::artmsQuantification with default settings. All peptides containing the same set of phosphorylated sites were grouped and quantified together into phosphorylation site groups and equalize median normalization was performed across runs to control for differences in sample preparation. Next, we performed statistical tests in MSstats to compare phosphopeptide intensities between stimulated and control conditions for each time point. We compared each stimulation condition to its time-matched control and also directly compared stimulations to each other (i.e., Fibrin vs. iC3b). We used defaults for MSstats for adjusted P values, even in cases of n=2 biological replicates. By default, MSstats uses the Student’s t-test for P value calculation and the Benjamini–Hochberg method of FDR estimation to adjust P values. On average, we quantified between 2,000-6,000 phosphorylated peptides per sample, mapping to 300-3,000 different proteins per sample.

Kinase activity analysis of phosphoproteomics data. Fold changes from MSstats were reduced to a single fold change per site by choosing the fold change with the lowest P value (non-infinite log2-transformed fold change values) and used for kinase activity and enrichment analysis. Mus musculus phosphorylation sites were converted to their Homo sapiens orthologous sites. Orthologous pairs of gene identifiers between M.musculus and H.sapiens were downloaded from Ensembl using the BioMart. Ensembl gene identifiers were mapped to UniProt identifiers and orthologous pairs of sequences were aligned using the Needleman-Wunsch global alignment algorithm implemented using Biostrings v2.62.0 function pairwiseAlignment with default parameters in R. The resulting alignments were used to convert the sequence positions of phosphorylations in M.musculus to positions in H.sapiens protein sequences, if possible. Kinase activities were estimated using known kinase–substrate relationships and inferred as a z-score calculated using the mean log2FC of phosphorylated substrates for each kinase in terms of standard error ($z=(M-u)/s.e.$), comparing fold changes in phosphosite measurements of the known substrates against the overall distribution of fold changes across the sample. P values were calculated using a two-tailed z-test. We collected substrate annotations for 400 kinases with available data. Kinases with 2 or more measured substrates were considered as predicted kinases (Supplementary Table 11).

Network reconstruction and enrichment analysis of phosphoproteomics data. Proteins with changes in phosphorylation state were selected based on an FDR threshold of 0.05. To compare phosphorylation status at two time points, 1 h and 3 h, protein-phosphorylation site pairs that

were found to be significant at least at one time point were maintained. After filtering, iC3b resulted in 44 phosphoproteins, and fibrin resulted in 68 phosphoproteins. To investigate the functional relatedness of proteins, STRING database was queried using the network analysis tool Cytoscape. Proteins with STRING interaction scores higher than 0.4 were connected by edges with widths and opacities reflecting the score level. Phosphorylation state changes were visualized using Omics Visualizer as two outer ring circles, with each layer representing phosphorylation at 1 h and 3 h. In order to enhance the signal for enrichment analysis, we also included up to ten additional nodes (proteins) identified by the STRING database as functionally related to our phosphoproteins using the stringApp. Final results are filtered based on an FDR threshold of 0.05 and redundant results were removed using a redundancy cutoff of 0.5. Two significant Gene Ontology (GO) Biological Process terms were selected and visualized as node fill colors. STRING-provided proteins and unconnected proteins were removed for visualization.

For manuscripts utilizing custom algorithms or software that are central to the research but not yet described in published literature, software must be made available to editors and reviewers. We strongly encourage code deposition in a community repository (e.g. GitHub). See the Nature Portfolio guidelines for submitting code & software for further information.

Data

Policy information about availability of data

All manuscripts must include a data availability statement. This statement should provide the following information, where applicable:

- Accession codes, unique identifiers, or web links for publicly available datasets
- A description of any restrictions on data availability
- For clinical datasets or third party data, please ensure that the statement adheres to our policy

The scRNA-seq and bulk RNA-seq datasets are deposited in the Genome Expression Omnibus under the SuperSeries accession number GSE229376. A searchable web resource of the microglia and BMDM ligand-activation scRNA-seq data are available at https://toxseq.shinyapps.io/ligand_activation/ and the single-cell 5XFAD Tox-seq data at https://toxseq.shinyapps.io/5xfad_toxseq/. The mass spectrometry proteomics data have been deposited to the ProteomeXchange Consortium via the PRIDE partner repository with the dataset identifier PXD021230. The protein interaction networks have been made available through NDEx at https://doi:10.18119/N9CK5Z and https://doi:10.18119/N9H89M.

Field-specific reporting

Please select the one below that is the best fit for your research. If you are not sure, read the appropriate sections before making your selection.

☒ Life sciences ☐ Behavioural & social sciences ☐ Ecological, evolutionary & environmental sciences

For a reference copy of the document with all sections, see nature.com/documents/nr-reporting-summary-flat.pdf

Life sciences study design

All studies must disclose on these points even when the disclosure is negative.

Sample size	No statistical methods were used to pre-determine sample sizes but sample sizes are similar to our previous publications (Ryu et al., Nat Immunol., 19, 1212-1223 (2018); Mendiola et al. Nat Immunol., 21, 513-524 (2020).
Data exclusions	From bulk RNA-seq experiment, three samples were removed that did not pass RNA and cDNA library quality control testing. One sample was removed due to large deviation on PCA and poor sequence alignment. For phosphoproteomic data, two sample outliers in intensities and peptide detections were discarded prior to quantitative analysis: Fibrin 1 h (PRIDE sample ID FU20180420-23) and one iC3b 1 h (PRIDE sample ID FU20180420-05) samples. No samples or animals were excluded from any other analyses.
Replication	The number of experimental repeats is detailed at the bottom of each legend for each figure. All attempts at replication following the protocols described in the methods were successful.
Randomization	Mice were randomized and blindly coded for group assignment and data collection for IHC and ICC experiments. For in vivo stereotactic plasma injections, mice were randomized and blindly coded for group assignment and data collection. For all scRNA-seq experiments, mice were randomized by sex and genotype prior to sample preparation.
Blinding	Stereotactic surgery was performed blinded to the experimental groups. For IHC experiments, image acquisition and quantification was performed by observers blinded to experimental conditions. Images were quantified independently by two blinded observers.

Reporting for specific materials, systems and methods

We require information from authors about some types of materials, experimental systems and methods used in many studies. Here, indicate whether each material, system or method listed is relevant to your study. If you are not sure if a list item applies to your research, read the appropriate section before selecting a response.

Materials & experimental systems

n/a	Involved in the study
☐	☒ Antibodies
☐	☒ Eukaryotic cell lines
☒	☐ Palaeontology and archaeology
☐	☒ Animals and other organisms
☒	☐ Human research participants
☒	☐ Clinical data
☒	☐ Dual use research of concern

Methods

n/a	Involved in the study
☒	☐ ChIP-seq
☐	☒ Flow cytometry
☒	☐ MRI-based neuroimaging

Antibodies

Antibodies used	Purified mouse IgG2b isotype control (clone MPC-11, BioXcell). IHC: GP91phox (1:150 dilution; 53, BD Biosciences), rabbit anti-IBA1 (1:500; 019-19741, Wako), goat anti-APOE (1:50; AB947, MilliporeSigma), p-PXN (1:500, rabbit polyclonal, #PAB7932, Abnova), p-MEK2 (1:500, rabbit polyclonal, #28955-1-AP, Thermo Fisher Scientific), and Alexa 647, 488 and Cy3 (1:500 dilution; Jackson ImmunoResearch). Immunoblots: p-NCF2 (1:1000, rabbit polyclonal, #PA5-105094, Thermo Fisher Scientific), p-PXN (1:1000, rabbit polyclonal, #PAB7932, Abnova), paxillin (1:1000, rabbit monoclonal, #ab32115, Abcam), p-MEK2 (1:1000, rabbit polyclonal, #28955-1-AP, Thermo Fisher Scientific), MEK1/2 (1:10,000, rabbit monoclonal, #ab178876, Abcam), and GAPDH (1:10,000, rabbit monoclonal, #2118, Cell Signaling Technology). FACS: CD11b (M1/70; Biolegend) and CD45 (30-F11; Biolegend).
Validation	All antibodies used in this study are from commercial sources and have been validated by the vendors and previous studies done by our lab or other labs. IgG2b, CD11b, CD45, GP91phox, IBA1, GAPDH were previously validated in our publications Ryu et al., Nat Commun 6, (2015); Ryu et al., Nat Immunol., 19, 1212-1223 (2018); Mendiola et al. Nat Immunol., 21, 513-524 (2020). APOE, manufactures information on validation not available but was used in Margeta et al Immunity 55, 1627-1644.e7 (2022); p-MEK2, manufactures information on validation not available, p-PAK, manufactures information on validation not available; MEK1/2, manufactures information on validation not available but was used in Ben-Addi et al Proc Natl Acad Sci USA 111, E2394-403 (2014); p-NCF2, manufactures information on validation not available. Paxillin was validated in KO cell lines (Abcam).

Eukaryotic cell lines

Policy information about cell lines

Cell line source(s)	RAW 264.7 mouse macrophage cell line was obtained from ATCC and cultured in DMEM supplemented with 10% FBS.
Authentication	RAW 264.7 macrophages were not authenticated
Mycoplasma contamination	The cell line was tested and was free from mycoplasma contamination.
Commonly misidentified lines (See ICLAC register)	No commonly misidentified cell lines were used.

Animals and other organisms

Policy information about studies involving animals; ARRIVE guidelines recommended for reporting animal research

Laboratory animals	Male and Female C57BL/6J and B6.Cg-Tg(APPSwFlLon,PSEN1*M146L*L286V) 6799Vas/Mmjax (5XFAD) mice were purchased from the Jackson Laboratory. Fga–/– and Fgg390-396A mice were obtained from Dr. Jay Degen (University of Cincinnati, OH, USA). 5XFAD mice were crossed with Fgg390-396A mice to generate 5XFAD:Fgg390-396A mice. Male and female mice were used in this study. Sprague-Dawley female rats with litters were purchased from Charles River. Animals were housed under IACUC guidelines in a temperature and humidity-controlled facility with 12 h light–12 h dark cycle and ad libitum feeding.
Wild animals	The study did not involve wild animals
Field-collected samples	The study did not involve samples collected from the field
Ethics oversight	All animal protocols were approved by the Committee of Animal Research at the University of California, San Francisco, and were in accordance with the National Institutes of Health guidelines.

Note that full information on the approval of the study protocol must also be provided in the manuscript.

Flow Cytometry

Plots

Confirm that:

☒ The axis labels state the marker and fluorochrome used (e.g. CD4-FITC).

☒ The axis scales are clearly visible. Include numbers along axes only for bottom left plot of group (a 'group' is an analysis of identical markers).

☒ All plots are contour plots with outliers or pseudocolor plots.

☒ A numerical value for number of cells or percentage (with statistics) is provided.

Methodology

Sample preparation	Mice were perfused with ice-cold phosphate-buffered saline (PBS) and cortical and hippocampal brain regions were carefully dissected from each animal and incubated with 1 mg mL-1 collagenase D (Sigma-Aldrich) and 0.05 mg mL-1 DNase1 (Sigma-Aldrich) for 30 min at 37C. Myelin was depleted using the debris removal kit as described56. Myelin-cleared cell suspensions were treated for 5 min at 4C with Fc-block in BSA staining buffer (BD Biosciences) and then incubated for 30 min at 4C with CD11b APC-Cy7 (M1/70) antibodies. For ROS and live cell labeling, cells were incubated with 10 uM 2’,7’-dichlorofluorescein diacetate (DCFDA) at 4C for 30 min and then incubated with 1 uM Sytox blue live/dead stain for 5 min at 4C. Using a FACSAria II (BD Biosciences), cells were sorting into live Sytox blue–CD11b+ROS– and live Sytox blue–CD11b+ROS+ cell populations from 12m 5XFAD and WT mice. Sorted cells were kept on ice until all samples were collected and then resuspended in cold PBS supplemented with 2% FBS at 250 cells uL-1 and immediately processed for scRNA-seq
Instrument	ARIAII (BD)
Software	BD FACSDiva v8 and FlowJo software v10
Cell population abundance	When cells were sorted or enriched, the purity was confirmed by flow cytometry and routinely >95 %.
Gating strategy	5XFAD Tox-seq: Single cells were gated by FSC-A/SSC-A and then doublet discrimination was performed first by FSC-H/FSC-W followed by SSC-H/SSC-W. Next, live sytox- CD11b+ cells were gated for, then cells were sorted based on ROS- and ROS+ (assessed by DCFDA).<br><br>Bulk RNA-seq experiment: Single cells were gated by FSC-A/SSC-A and then doublet discrimination was performed first by FSC-H/FSC-W followed by SSC-H/SSC-W. Then, microglia cells were gated by CD45loCD11b+ expression.

☒ Tick this box to confirm that a figure exemplifying the gating strategy is provided in the Supplementary Information.
