# Supplementary material for: Defining blood-induced microglia functions in neurodegeneration through multiomic profiling
Source: Nat Immunol. 2023 Jun 8;24(7):1173–87. doi: 10.1038/s41590-023-01522-0 (PMC10307624; doi:10.1038/s41590-023-01522-0)

Full, uncropped immunoblots for Fig. 4.

Fig. 4f uncropped and unprocessed immunoblots

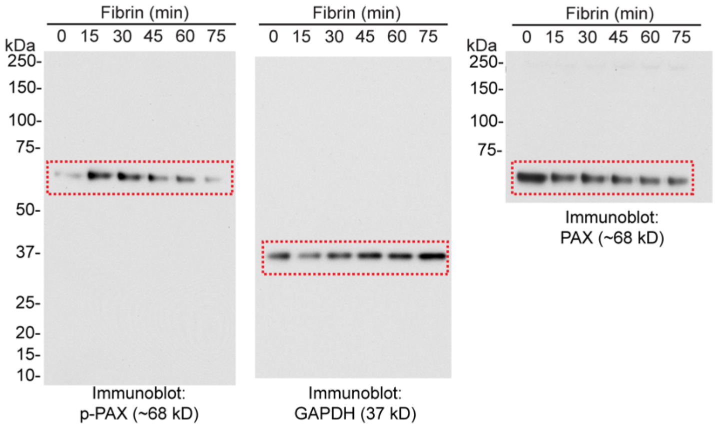

Fig. 4g uncropped and unprocessed immunoblots

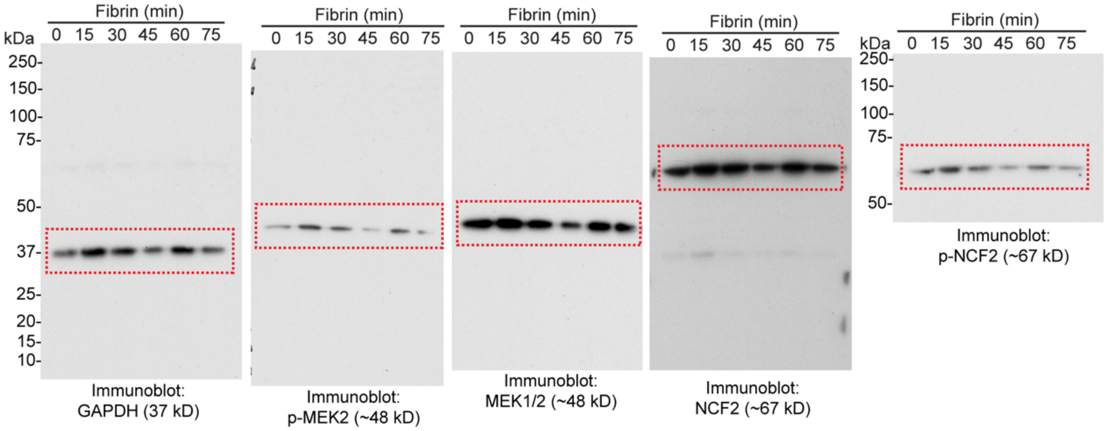

Fig. 4i uncropped and unprocessed immunoblots

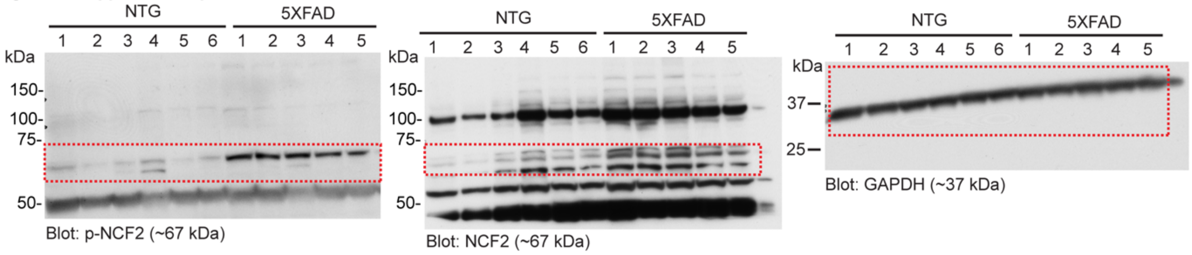

Supplement: Source Data Fig. 4 — Unprocessed immunoblots. [file 41590_2023_1522_MOESM6_ESM.pdf]
